# Supplementary material for: Fuzzy clustering for the within-season estimation of cotton phenology
Source: PLoS One. 2023 Mar 8;18(3):e0282364. doi: 10.1371/journal.pone.0282364 (PMC9994758; doi:10.1371/journal.pone.0282364)
Supplement: S1 Table — With (I) we show the cumulative integrals of the VIs. max_soil refers to the cumulative maximum soil temperature, max_surf to the cumulative maximum surface temperature and wkappa to the linear weighted kappa coefficient. The last five columns refer to the performance metrics. (PDF) [file pone.0282364.s001.pdf]

S1 Table. The feature sets of the top 82 FCM models with size 8 or 9 features. With (I) we show the cumulative integrals of the VIs. max_soil refers to the cumulative maximum soil temperature, max_surf to the cumulative maximum surface temperature and wkappa to the linear weighted kappa coefficient. The last five columns refer to the performance metrics.

feature1	feature2	feature3	feature4	feature5	feature6	feature7	feature8	feature9	maxdiff-0	maxdiff-1	maxdiff-2	kappa	wkappa
cos_doy	sin_doy	savi	wdrvi(I)	ndwi(I)	gdd	max_soil	max_surf	-	0.5813	0.864	0.9947	0.5323	0.8884
cos_doy	sin_doy	gvmi	wdrvi(I)	ndwi(I)	gdd	max_soil	max_surf	-	0.5653	0.8773	0.9947	0.5148	0.8883
cos_doy	sin_doy	evi	savi	wdrvi(I)	psri(I)	gdd	max_surf	-	0.552	0.8693	0.9973	0.504	0.8846
cos_doy	sin_doy	gvmi	savi	wdrvi(I)	psri(I)	ndwi(I)	gdd	max_soil	0.5547	0.888	0.9893	0.5031	0.8875
cos_doy	sin_doy	gvmi	evi	wdrvi(I)	gdd	max_soil	max_surf	-	0.5493	0.8747	0.9973	0.4994	0.8843
cos_doy	sin_doy	evi	savi	wdrvi(I)	gdd	max_soil	max_surf	-	0.5493	0.8667	0.9973	0.4975	0.8836
cos_doy	sin_doy	evi	savi	wdrvi(I)	psri(I)	ndwi(I)	gdd	max_surf	0.5467	0.872	0.9787	0.4934	0.8799
cos_doy	sin_doy	gvmi	savi	wdrvi(I)	psri(I)	gdd	max_surf	-	0.5413	0.88	0.984	0.4929	0.8825
cos_doy	sin_doy	gvmi	wdrvi(I)	psri(I)	gdd	max_soil	max_surf	-	0.544	0.8667	0.9973	0.4912	0.8816
cos_doy	sin_doy	evi	savi	psri(I)	ndwi(I)	gdd	max_soil	-	0.544	0.856	0.992	0.491	0.8782
cos_doy	sin_doy	evi	savi	ndwi(I)	gdd	max_soil	max_surf	-	0.5387	0.8907	0.9973	0.4887	0.8861
cos_doy	sin_doy	gvmi	evi	savi	wdrvi(I)	gdd	max_soil	-	0.5413	0.88	0.984	0.4883	0.8822
cos_doy	sin_doy	gvmi	wdrvi(I)	psri(I)	ndwi(I)	gdd	max_soil	max_surf	0.5413	0.864	0.984	0.4879	0.8777
cos_doy	sin_doy	evi	savi	psri(I)	ndwi(I)	gdd	max_surf	-	0.5413	0.8747	0.9867	0.4872	0.8807
cos_doy	sin_doy	evi	wdrvi(I)	ndwi(I)	gdd	max_soil	max_surf	-	0.5387	0.8293	0.9787	0.4867	0.8699
cos_doy	sin_doy	gvmi	evi	wdrvi(I)	ndwi(I)	gdd	max_soil	max_surf	0.536	0.88	0.984	0.4863	0.8805
cos_doy	sin_doy	gvmi	evi	wdrvi(I)	psri(I)	gdd	max_soil	-	0.536	0.856	0.9867	0.4858	0.8758
cos_doy	sin_doy	evi	savi	wdrvi(I)	ndwi(I)	gdd	max_soil	-	0.536	0.8427	0.9813	0.4848	0.8722
cos_doy	sin_doy	evi	savi	wdrvi(I)	psri(I)	gdd	max_soil	-	0.536	0.8667	0.9947	0.4844	0.8803
cos_doy	sin_doy	evi	savi	wdrvi(I)	ndwi(I)	gdd	max_soil	max_surf	0.5333	0.904	1.0	0.4832	0.8884
cos_doy	sin_doy	evi	savi	wdrvi(I)	psri(I)	ndwi(I)	gdd	max_soil	0.5333	0.8613	0.9787	0.4827	0.8753
cos_doy	sin_doy	gvmi	evi	ndwi(I)	gdd	max_soil	max_surf	-	0.536	0.88	0.9973	0.482	0.8831
cos_doy	sin_doy	gvmi	evi	wdrvi(I)	ndwi(I)	gdd	max_surf	-	0.5333	0.872	0.9947	0.4815	0.88
cos_doy	sin_doy	gvmi	evi	savi	wdrvi(I)	psri(I)	gdd	max_soil	0.5307	0.9173	0.9893	0.4784	0.889
cos_doy	sin_doy	evi	savi	psri(I)	ndwi(I)	gdd	max_soil	max_surf	0.528	0.896	0.9947	0.477	0.8844
cos_doy	sin_doy	savi	wdrvi(I)	psri(I)	ndwi(I)	gdd	max_soil	max_surf	0.528	0.8693	0.992	0.4755	0.8785
cos_doy	sin_doy	gvmi	evi	psri(I)	ndwi(I)	gdd	max_soil	-	0.5253	0.8613	1.0	0.4753	0.8777
cos_doy	sin_doy	savi	wdrvi(I)	psri(I)	ndwi(I)	gdd	max_soil	-	0.5253	0.872	0.9867	0.4752	0.8774
cos_doy	sin_doy	gvmi	evi	wdrvi(I)	psri(I)	gdd	max_soil	max_surf	0.528	0.8827	0.9947	0.475	0.8806
cos_doy	sin_doy	gvmi	savi	wdrvi(I)	ndwi(I)	gdd	max_surf	-	0.528	0.8747	0.9627	0.4737	0.8748
cos_doy	sin_doy	gvmi	wdrvi(I)	psri(I)	ndwi(I)	gdd	max_surf	-	0.5227	0.8453	0.984	0.4715	0.8706
cos_doy	sin_doy	gvmi	savi	psri(I)	ndwi(I)	gdd	max_soil	-	0.52	0.8853	0.992	0.471	0.882
cos_doy	sin_doy	gvmi	evi	wdrvi(I)	psri(I)	gdd	max_surf	-	0.5227	0.848	0.9947	0.4709	0.8731
cos_doy	sin_doy	gvmi	evi	savi	psri(I)	ndwi(I)	gdd	max_soil	0.5253	0.8987	1.0	0.4701	0.8856
cos_doy	sin_doy	gvmi	savi	wdrvi(I)	ndwi(I)	gdd	max_soil	-	0.52	0.8773	0.9787	0.4695	0.8771
cos_doy	sin_doy	evi	savi	wdrvi(I)	psri(I)	gdd	max_soil	max_surf	0.52	0.8667	0.9973	0.4687	0.8775
cos_doy	sin_doy	gvmi	savi	psri(I)	ndwi(I)	gdd	max_soil	max_surf	0.5173	0.904	1.0	0.468	0.8852
cos_doy	sin_doy	gvmi	savi	psri(I)	ndwi(I)	gdd	max_surf	-	0.52	0.8773	0.9813	0.4675	0.8764
cos_doy	sin_doy	gvmi	savi	ndwi(I)	gdd	max_soil	max_surf	-	0.52	0.896	0.9947	0.4671	0.8836
cos_doy	sin_doy	gvmi	evi	savi	wdrvi(I)	psri(I)	gdd	max_surf	0.5173	0.88	0.9893	0.4653	0.8781
cos_doy	sin_doy	gvmi	evi	savi	wdrvi(I)	psri(I)	gdd	-	0.5173	0.8667	0.9893	0.4649	0.8742
cos_doy	sin_doy	gvmi	evi	psri(I)	ndwi(I)	gdd	max_surf	-	0.5173	0.856	0.992	0.4647	0.8727
cos_doy	sin_doy	evi	psri(I)	ndwi(I)	gdd	max_soil	max_surf	-	0.5147	0.8453	0.984	0.4638	0.8693
cos_doy	sin_doy	savi	wdrvi(I)	psri(I)	ndwi(I)	gdd	max_surf	-	0.512	0.8507	0.976	0.4622	0.8691
cos_doy	sin_doy	gvmi	evi	savi	wdrvi(I)	ndwi(I)	gdd	-	0.5173	0.8453	0.9573	0.462	0.8657
cos_doy	sin_doy	evi	wdrvi(I)	psri(I)	gdd	max_soil	max_surf	-	0.512	0.8107	0.9787	0.4614	0.8601
cos_doy	sin_doy	gvmi	savi	wdrvi(I)	psri(I)	ndwi(I)	gdd	-	0.5147	0.848	0.96	0.4608	0.8654
cos_doy	sin_doy	gvmi	evi	savi	wdrvi(I)	ndwi(I)	gdd	max_soil	0.512	0.872	0.976	0.4603	0.8737
cos_doy	sin_doy	evi	savi	wdrvi(I)	ndwi(I)	gdd	max_surf	-	0.512	0.8613	0.9867	0.4593	0.8731
cos_doy	sin_doy	savi	psri(I)	ndwi(I)	gdd	max_soil	max_surf	-	0.5093	0.864	1.0	0.4583	0.8745
cos_doy	sin_doy	gvmi	wdrvi(I)	psri(I)	ndwi(I)	gdd	max_soil	-	0.512	0.872	0.984	0.4569	0.8739
cos_doy	sin_doy	gvmi	evi	wdrvi(I)	psri(I)	ndwi(I)	gdd	-	0.5093	0.8347	0.9707	0.4556	0.8622
cos_doy	sin_doy	gvmi	evi	savi	wdrvi(I)	gdd	max_surf	-	0.5093	0.8827	0.9893	0.4551	0.8775
cos_doy	sin_doy	gvmi	evi	psri(I)	gdd	max_soil	max_surf	-	0.504	0.8587	0.9973	0.4547	0.8719
cos_doy	sin_doy	gvmi	savi	wdrvi(I)	psri(I)	gdd	max_soil	-	0.504	0.8747	0.9893	0.454	0.8749
cos_doy	sin_doy	gvmi	evi	savi	wdrvi(I)	gdd	max_soil	max_surf	0.504	0.88	0.9947	0.4535	0.8771
cos_doy	sin_doy	gvmi	evi	savi	ndwi(I)	gdd	max_soil	max_surf	0.504	0.8773	0.9947	0.4531	0.8759
cos_doy	sin_doy	gvmi	evi	savi	wdrvi(I)	psri(I)	ndwi(I)	gdd	0.5067	0.8587	0.96	0.4512	0.8666
cos_doy	sin_doy	gvmi	evi	wdrvi(I)	psri(I)	ndwi(I)	gdd	max_soil	0.504	0.8427	0.9813	0.4506	0.866
cos_doy	sin_doy	evi	wdrvi(I)	psri(I)	ndwi(I)	gdd	max_soil	max_surf	0.504	0.8187	0.9867	0.4498	0.8621
cos_doy	sin_doy	evi	savi	psri(I)	gdd	max_soil	max_surf	-	0.5013	0.8693	1.0	0.448	0.8756
cos_doy	sin_doy	gvmi	evi	savi	psri(I)	gdd	max_soil	-	0.496	0.8693	1.0	0.4469	0.8743
cos_doy	sin_doy	gvmi	evi	savi	ndwi(I)	gdd	max_soil	-	0.5013	0.9067	0.992	0.4468	0.8806
cos_doy	sin_doy	gvmi	evi	wdrvi(I)	psri(I)	ndwi(I)	gdd	max_surf	0.5013	0.8533	0.9893	0.4468	0.8691
cos_doy	sin_doy	gvmi	evi	wdrvi(I)	ndwi(I)	gdd	max_soil	-	0.4987	0.8667	0.9893	0.4464	0.8729
cos_doy	sin_doy	gvmi	evi	savi	psri(I)	ndwi(I)	gdd	-	0.4987	0.8693	0.9813	0.4454	0.8709
cos_doy	sin_doy	gvmi	evi	psri(I)	ndwi(I)	gdd	max_soil	max_surf	0.496	0.88	0.9973	0.4445	0.8757
cos_doy	sin_doy	savi	wdrvi(I)	psri(I)	gdd	max_soil	max_surf	-	0.4933	0.8667	0.9973	0.4444	0.8725
cos_doy	sin_doy	gvmi	savi	wdrvi(I)	ndwi(I)	gdd	max_soil	max_surf	0.4933	0.8907	0.9867	0.4442	0.8757
cos_doy	sin_doy	gvmi	evi	savi	psri(I)	ndwi(I)	gdd	max_surf	0.488	0.8853	0.9893	0.435	0.8736
cos_doy	sin_doy	evi	savi	wdrvi(I)	psri(I)	ndwi(I)	gdd	-	0.488	0.856	0.9627	0.4328	0.862
cos_doy	sin_doy	gvmi	savi	wdrvi(I)	psri(I)	ndwi(I)	gdd	max_surf	0.4853	0.888	0.9813	0.4325	0.872
cos_doy	sin_doy	evi	wdrvi(I)	psri(I)	ndwi(I)	gdd	max_soil	-	0.4853	0.8133	0.9787	0.4319	0.8549
cos_doy	sin_doy	gvmi	evi	savi	psri(I)	gdd	max_soil	max_surf	0.4827	0.888	0.9947	0.4314	0.8747
cos_doy	sin_doy	gvmi	evi	savi	wdrvi(I)	ndwi(I)	gdd	max_surf	0.4827	0.8667	0.9733	0.4301	0.8666
cos_doy	sin_doy	gvmi	psri(I)	ndwi(I)	gdd	max_soil	max_surf	-	0.48	0.8587	1.0	0.4276	0.8685
cos_doy	sin_doy	gvmi	evi	savi	psri(I)	gdd	max_surf	-	0.4773	0.888	0.9947	0.4274	0.8735
cos_doy	sin_doy	evi	wdrvi(I)	psri(I)	ndwi(I)	gdd	max_surf	-	0.4827	0.84	0.976	0.4259	0.8598
cos_doy	sin_doy	gvmi	savi	psri(I)	gdd	max_soil	max_surf	-	0.4773	0.9013	1.0	0.4258	0.8768
cos_doy	sin_doy	gvmi	savi	wdrvi(I)	psri(I)	gdd	max_soil	max_surf	0.4747	0.88	0.9893	0.4233	0.8711
cos_doy	sin_doy	gvmi	evi	savi	ndwi(I)	gdd	max_surf	-	0.4773	0.8933	0.9893	0.4225	0.8729
cos_doy	sin_doy	gvmi	savi	wdrvi(I)	gdd	max_soil	max_surf	-	0.472	0.8827	0.9973	0.4196	0.8714
