# Supplementary material for: Fuzzy clustering for the within-season estimation of cotton phenology
Source: PLoS One. 2023 Mar 8;18(3):e0282364. doi: 10.1371/journal.pone.0282364 (PMC9994758; doi:10.1371/journal.pone.0282364)
Supplement: Attachment — Submitted filename: PONE-D-22-14150_r1.pdf [file pone.0282364.s002.pdf]

PLOS ONE

Fuzzy clustering for the within-season estimation of cotton phenology

--Manuscript Draft--

**Manuscript Number:**	PONE-D-22-14150
**Article Type:**	Research Article
**Full Title:**	Fuzzy clustering for the within-season estimation of cotton phenology
**Short Title:**	Fuzzy clustering for phenology estimation
**Corresponding Author:**	Vasileios Sitokonstantinou, M.Sc.<br>National Observatory of Athens: Ethnikon Asteroskopeion Athenon<br>Athens, Select an option… GREECE
**Keywords:**	Crop phenology; numerical weather predictions; satellite image time-series; Sentinel-2; smart farming,; fuzzy clustering; field visits
**Abstract:**	Crop phenology is crucial information for crop yield estimation and agricultural management. Traditionally, phenology has been observed from the ground; however earth observation, weather and soil data have been used to capture the physiological growth of crops. In this work, we propose a new approach for the within-season phenology estimation for cotton at the field level. For this, we exploit a variety of earth observation vegetation indices (derived from Sentinel-2) and numerical simulations of atmospheric and soil parameters. Our method is unsupervised to address the ever-present problem of sparse and scarce ground truth data that makes most supervised alternatives impractical in real-world scenarios. We applied fuzzy c-means clustering to identify the principal phenological stages of cotton and then used the cluster membership weights to further predict the transitional phases between adjacent stages. In order to evaluate our models, we collected hundreds of crop growth ground observations in Orchomenos, Greece. We introduced a new collection protocol, assigning up to two phenology labels that represent the primary and secondary growth stage in the field and thus indicate when stages are transitioning. Our model was tested against a baseline model that allowed to isolate the random agreement and evaluate its true competence. The results showed that our model considerably outperforms the baseline one, which is promising considering the unsupervised nature of the approach. The limitations and the relevant future work are thoroughly discussed. The ground observations are formatted in an ready-to-use dataset and will be available at https://github.com/Agri-Hub/cotton-phenology-dataset upon publication.
**Order of Authors:**	Vasileios Sitokonstantinou
	Alkiviadis Koukos
	Ilias Tsoumas
	Charalampos Kontoes
	Nikolaos S. Bartsotas
	Vassilia Karathanassi
**Additional Information:**	
**Question**	**Response**
**Financial Disclosure**<br><br>Enter a financial disclosure statement that describes the sources of funding for the work included in this submission. Review the submission guidelines for detailed requirements. View published research articles from *PLOS ONE* for specific examples.	This work has been supported by the e-shape project (https://e-shape.eu/), which has been funded from the European Union's Horizon 2020 research and innovation programme under grant agreement 820852. There was no additional external funding received for this study. The funders had no role in study design, data collection and analysis, decision to publish, or preparation of the manuscript.

Powered by Editorial Manager® and ProduXion Manager® from Aries Systems Corporation

This statement is required for submission and **will appear in the published article** if the submission is accepted. Please make sure it is accurate.<br><br>**Unfunded studies**<br>Enter: *The author(s) received no specific funding for this work.*<br><br>**Funded studies**<br>Enter a statement with the following details:<br>• Initials of the authors who received each award<br>• Grant numbers awarded to each author<br>• The full name of each funder<br>• URL of each funder website<br>• Did the sponsors or funders play any role in the study design, data collection and analysis, decision to publish, or preparation of the manuscript?<br>• **NO** - Include this sentence at the end of your statement: *The funders had no role in study design, data collection and analysis, decision to publish, or preparation of the manuscript.*<br>• **YES** - Specify the role(s) played.<br><br>* typeset	
**Competing Interests**<br><br>Use the instructions below to enter a competing interest statement for this submission. On behalf of all authors, disclose any competing interests that could be perceived to bias this work—acknowledging all financial support and any other relevant financial or non-financial competing interests.<br><br>This statement is required for submission and **will appear in the published article** if the submission is accepted. Please make sure it is accurate and that any funding sources listed in your Funding Information later in the submission form are also declared in your Financial Disclosure statement.<br><br>View published research articles from	The authors have declared that no competing interests exist.

PLOS ONE for specific examples.<br><br>**NO authors have competing interests**<br><br>Enter: *The authors have declared that no competing interests exist.*<br><br>**Authors with competing interests**<br><br>Enter competing interest details beginning with this statement:<br><br>*I have read the journal's policy and the authors of this manuscript have the following competing interests: [insert competing interests here]*<br><br>* typeset	
**Ethics Statement**<br><br>Enter an ethics statement for this submission. This statement is required if the study involved:<br><br>• Human participants<br>• Human specimens or tissue<br>• Vertebrate animals or cephalopods<br>• Vertebrate embryos or tissues<br>• Field research<br><br>Write "N/A" if the submission does not require an ethics statement.<br><br>General guidance is provided below. Consult the submission guidelines for detailed instructions. **Make sure that all information entered here is included in the Methods section of the manuscript.**	Permission to conduct field visits and collect ground observations was granted by the Farmers' Cooperative of Orchomenos (GREECE).

**Format for specific study types**<br><br>**Human Subject Research (involving human participants and/or tissue)**<br>• Give the name of the institutional review board or ethics committee that approved the study<br>• Include the approval number and/or a statement indicating approval of this research<br>• Indicate the form of consent obtained (written/oral) or the reason that consent was not obtained (e.g. the data were analyzed anonymously)<br><br>**Animal Research (involving vertebrate animals, embryos or tissues)**<br>• Provide the name of the Institutional Animal Care and Use Committee (IACUC) or other relevant ethics board that reviewed the study protocol, and indicate whether they approved this research or granted a formal waiver of ethical approval<br>• Include an approval number if one was obtained<br>• If the study involved *non-human primates*, add *additional details* about animal welfare and steps taken to ameliorate suffering<br>• If anesthesia, euthanasia, or any kind of animal sacrifice is part of the study, include briefly which substances and/or methods were applied<br><br>**Field Research**<br><br>Include the following details if this study involves the collection of plant, animal, or other materials from a natural setting:<br>• Field permit number<br>• Name of the institution or relevant body that granted permission	
**Data Availability**<br><br>Authors are required to make all data underlying the findings described fully available, without restriction, and from the time of publication. PLOS allows rare exceptions to address legal and ethical concerns. See the PLOS Data Policy and FAQ for detailed information.	Yes - all data are fully available without restriction

A Data Availability Statement describing where the data can be found is required at submission. Your answers to this question constitute the Data Availability Statement and **will be published in the article**, if accepted.<br><br>**Important:** Stating 'data available on request from the author' is not sufficient. If your data are only available upon request, select 'No' for the first question and explain your exceptional situation in the text box.<br><br>Do the authors confirm that all data underlying the findings described in their manuscript are fully available without restriction?	
**Describe where the data may be found in full sentences. If you are copying our sample text, replace any instances of XXX with the appropriate details.**<br><br>• If the data are **held or will be held in a public repository**, include URLs, accession numbers or DOIs. If this information will only be available after acceptance, indicate this by ticking the box below. For example: *All XXX files are available from the XXX database (accession number(s) XXX, XXX.*).<br>• If the data are all contained **within the manuscript and/or Supporting Information files**, enter the following: *All relevant data are within the manuscript and its Supporting Information files.*<br>• If neither of these applies but you are able to provide **details of access elsewhere**, with or without limitations, please do so. For example:<br><br>*Data cannot be shared publicly because of [XXX]. Data are available from the XXX Institutional Data Access / Ethics Committee (contact via XXX) for researchers who meet the criteria for access to confidential data.*<br><br>*The data underlying the results presented in the study are available from (include the name of the third party*	This work's dataset, which includes all ground images and observations, will be available at https://github.com/Agri-Hub/cotton-phenology-dataset upon publication. A sample is already available, along with pertinent text that explains how to use the dataset and how it was collected. Upon publication the dataset will be published on dryads and the doi will be linked with the publication.

and contact information or URL).<br>• This text is appropriate if the data are owned by a third party and authors do not have permission to share the data.<br><br>* typeset	
Additional data availability information:	Tick here if the URLs/accession numbers/DOIs will be available only after acceptance of the manuscript for publication so that we can ensure their inclusion before publication.

Powered by Editorial Manager® and ProduXion Manager® from Aries Systems Corporation

Manuscript

Click here to access/download;Manuscript;PLOS_ONE_Fuzzy_CLustering.pdf

Fuzzy clustering for the within-season estimation of cotton phenology

Vasileios Sitokonstantinou[1,2*], Alkiviadis Koukos[1], Ilias Tsoumas[1], Nikolaos S. Bartsotas[1], Charalampos Kontoes[1], Vassilia Karathanassi[2]

1 National Observatory of Athens, IAASARS, BEYOND Centre of EO Research and Satellite Remote Sensing, Athens, Greece, **2** Laboratory of Remote Sensing, National Technical University of Athens, Athens, Greece.

* vsito@noa.gr

Abstract

Crop phenology is crucial information for crop yield estimation and agricultural management. Traditionally, phenology has been observed from the ground; however earth observation, weather and soil data have been used to capture the physiological growth of crops. In this work, we propose a new approach for the within-season phenology estimation for cotton at the field level. For this, we exploit a variety of earth observation vegetation indices (derived from Sentinel-2) and numerical simulations of atmospheric and soil parameters. Our method is unsupervised to address the ever-present problem of sparse and scarce ground truth data that makes most supervised alternatives impractical in real-world scenarios. We applied fuzzy c-means clustering to identify the principal phenological stages of cotton and then used the cluster membership weights to further predict the transitional phases between adjacent stages. In order to evaluate our models, we collected hundreds of crop growth ground observations in Orchomenos, Greece. We introduced a new collection protocol, assigning up to two phenology labels that represent the primary and secondary growth stage in the field and thus indicate when stages are transitioning. Our model was tested against a baseline model that allowed to isolate the random agreement and evaluate its true competence. The results showed that our model considerably outperforms the baseline one, which is promising considering the unsupervised nature of the approach. The limitations and the relevant future work are thoroughly discussed. The ground observations are formatted in an ready-to-use dataset and will be available at `https://github.com/Agri-Hub/cotton-phenology-dataset` upon publication.

Introduction

Crop phenology is key information for crop yield estimation and agricultural management and thereby actionable knowledge for the farmer, the agricultural consultant, the insurance company and the policy maker. Crop phenology is the physiological development of the plant from sowing to harvest. The precise and timely knowledge of the growth status of crops is crucial for estimating the yield early in the season, but also for taking prompt action on controlling the growth to i) maximize the production and ii) reduce the farming costs [1].

Crops' water needs are a function of the phenological stage. Using the example of cotton, which is the crop of interest in this study, there is higher water usage between

the flowering and early boll opening stages than in the emergence and late boll opening stages [2]. Irrigation can be interrupted on the onset of boll opening to stop the continuous growth of cotton and allow the photosynthetic carbohydrates to start contributing to the development of bolls and not the development of leaves and flowers [3]. Therefore, we need crop phenology information in order to make irrigation recommendations towards fully utilizing the expensive and often scarce water, and at the same time reduce water stress and its potential adverse effects on the yield [4]. Irrigation is one of the many examples of how phenology can benefit the agricultural practice management. Other examples include the precise fertilization, pest management and harvesting [1].

For many years, phenology has been observed from the ground, through field visits and in-situ sensors. These approaches however are expensive, time-consuming and lack spatial variability. To this end, space-borne and aerial remote sensing Vegetation Index (VI) time-series have been used to systematically monitor crop phenology over large geographic regions; often termed land surface phenology [1]. The freely available Sentinel-2 data offer optical imagery of high temporal and spatial resolution that introduced new opportunities for the large-scale and within-season monitoring of phenology [5, 6].

The recently published positioning papers [1, 7] and [8], identify the problem of remote phenology estimation as a fundamental one for the future of agriculture monitoring. Particularly, the authors underline the importance and the expected impact of within-season estimations at high spatial resolutions. Many of the related studies focus on the prediction of few principal phenological stages, failing to truly exploit the frequency of remote sensing data. This is mostly true because the required ground observations for training and/or evaluation are usually infrequent and lack spatial variability. This kind of ground observations are usually achieved with the use of networks of phenology stations or phenocams, which are always sparse and limited in number.

In the past two decades, there has been a number of related studies that focus on the estimation of vegetation phenology using both Earth Observation (EO) and weather data, under a wide variety of methodological frameworks. Initial approaches to the problem, many of which continue to develop to this day, offered after-season phenology estimations and were usually applied at large geographic scales using medium resolution imagery. The term after-season indicates that phenology is estimated after the crop is harvested and thus leverages the entire data time-series. This large-scale monitoring of the dynamics of phenology has been very popular in the scientific domains of ecology and climate change monitoring [6, 9–16]. Nevertheless, detailed information that is offered within the cultivation period is very important from the perspective of the farmer. Using timely and high spatial resolution phenology predictions, farmers can protect their yield and maximize their profit. On this account, there has been a number of studies that provide within-season phenology predictions at the field level [17–23]. Phenology estimation can be found in literature both as a classification and as a regression problem. For the first, the phenological cycle is broken down into distinct stages or classes that last for a given period of time [17–19]. On the other hand, phenology as a regression problem translates to predicting the day of the onset of key phenological phases [20, 21, 23, 24].

Each crop type has its own growth cycle and hence unique characteristics with respect to i) how phenology is affected by agro-climatic conditions and ii) how responsive are the space-borne or aerial EO data with respect to the various growth stages. There are crop types for which phenology and yield are highly correlated to the vegetation canopy we see from space, e.g., tobacco. This is not the case for other crops, especially those that bear fruits or bolls, where phenology and vegetation canopy are

not closely coupled. In literature, one can find many publications related to the estimation of phenology for rice, barley, soybean and maize [18, 19, 24–26]. In this study, however, we focus on the rather underrepresented crop type of cotton (Gossypium hirsutum L.). Cotton is a unique case with non-linear relationships between growth and the VIs that we have in our capacity to monitor it.

Phenology is affected by the effective solar radiation that enables photosynthesis, the soil properties, and many other agro-meteorological parameters. Indeed, in literature we can find older studies that use exclusively meteorological data [27–29] and other more recent ones that combine them with optical images [19, 21, 30]. Synthetic Aperture Radar (SAR) data, usually in combination with optical images, have been mostly used for estimating rice phenology, but also other crop types [18, 31]. The combination of Sentinel-2 and MODIS has been one of the most popular in the field. This is true because the Sentinel-2 missions offer data of high spatial resolution that enable information extraction at the field level, whereas MODIS data and their daily acquisitions allow for the generation of dense SITS [20, 22–24, 26, 32–34]. Other data sources found in literature include UAVs [35, 36] and in-field RGB sensors [37].

There are several published studies that employ supervised ML methods for land surface phenology. For instance, the authors in [19] have used Support Vector Machines (SVM) and Random Forests (RF) to integrate field, weather and satellite data for maize phenology monitoring. Furthermore, the authors in [21] and [38] have used traditional machine learning regressors to model plant phenology based on both satellite EO and gridded meteorological data. There have also been a few Deep Learning (DL) based approaches. One example is [32], where the authors explore the use of capsules, i.e., a group of neurons to address the issues of translation invariance prevalent in conventional CNNs, to learn the characteristic features of the phenological curves. There is also a number of methods that do not use ML. A few examples of such methods include dynamic multi-temporal modeling and Kalman filtering in [22], particle filtering in [23], sigmoid modeling in [33], first-derivative analysis in [31] and [20], and wavelet-based filtering and shape model fitting in [39].

In this work we exploit EO data (Sentinel-2), together with numerical simulations of atmospheric and soil parameters, to address the within-season phenology estimation for cotton at the field level. Even more, since ground truth data are scarce and expensive to collect, we predict phenological stages using clustering, in order to be truly useful in real world scenarios. We go beyond the estimation of principal phenological stages and additionally identify the fuzzy transitions between stages as individual metaclasses (two ranked labels). We focus on cotton that is a vital crop for the Greek economy and agricultural ecosystem, and even more has been significantly underrepresented in the phenology estimation literature. Finally, we developed and made publicly available a unique dataset of cotton growth ground observations, collected by an expert who performed hundreds of field visits in Orchomenos, Greece.

Materials and methods

Study Area and Field Campaign

Greece has the third largest production of cotton per person and is the number one producer in the European Union (EU). Cotton is extensively cultivated and is very important for the national economy, with Greece being the fifth largest exporter in the world. Unfortunately, however, there is no organized effort to record practice calendars and phenological observations. In Greece, cotton needs between 150 to 200 days in order to complete its phenological cycle. The duration depends on the cotton variety and the agro-climatic conditions [27].

For this study, cotton growth consists of six principal phenological stages. These refer to higher level groupings of the cotton growth micro-stages defined in the official manual for damage assessment of the Greek Agricultural Insurance Organization (ELGA) [40]. These groupings have been made after consulting experts in cotton growth. The first stage is Root Establishment (RE), referring to the period from sowing to the development of three leaves. This stage lasts between 15 to 30 days, but this can be greatly affected by weather conditions and particularly low temperatures that can slow down the process [27]. The second stage is Leaf Development (LD) and encompasses the period from the development of the fourth leaf to the appearance of the first squares. This usually takes between 35 to 45 days, but once again it is subject to weather conditions [41]. The third growth stage is Squaring (S) that includes the period between the formation of the first squares to the appearance of the first flowers. This stage takes between 15 to 30 days. Then the first flowers open with the onset of the Flowering (F) stage that lasts for 20 to 40 days. Then follows the Boll Development (BD) stage that takes 25 to 45 days until the start of leaf discoloration and the onset of Boll Opening (BO) that lasts roughly 10 to 20 days until harvest.

It should be noted that phenology is a dynamic variable and in principle can be described in great detail, going beyond these six principal crop growth stages. At any given instance, a cotton plant can be characterized by a combination of adjacent stages. For example, during the late flowering stage, a plant would have both flowers and cotton bolls, i.e., it would be transitioning to the BD stage. One of the most popular crop growth identification scales is the BBCH [42]. The BBCH scale makes use of a two digit representation, with the first digit referring to the principal growth stage and the second digit describing the secondary growth stage that corresponds to an ordinal number of percentage value. The BBCH scale ranges from 00 to 99. However, collecting ground observations of this detail is a challenging task because i) a large number of samples cannot be observed in near-daily frequency and ii) it is difficult, even for experts, to assign precise growth stages, especially when this decision needs to be aggregated at the field level.

In order to collect ground truth data that would allow us to evaluate the models of this study, an expert performed an extensive and intensive field campaign in Greece. The campaign took place during the growing season of 2021, from root establishment to boll opening, which for Greece extends between late April and early October. The expert followed the instructions that are summarized below:

- At least 15 visits per field (approx. 3 per month) during the growing period, including at least one visit per phenological stage.
- Ideally, visit the fields in the days that Sentinel-2 passes over. If this is logistically impossible, visit the fields maximum one day prior or after the Sentinel-2 pass.
- If it is cloudy, check the next Sentinel-2 pass, consult weather forecasts and decide if the inspection could be delayed for a few days or should happen irrespective of the cloud coverage.
- Walk with a zig-zag pattern for typical scouting through the field and inspect the growth status and how it varies in space.
- Decide on the phenological stage that best describes the majority of the plants in the field. If the field is in a transitioning phase between two phenological stages, mention both and decide which is the prevailing one, i.e., the primary stage.
- Decide on the percentage that is explained by the primary and the secondary stage

- Take a panoramic photo of the entire field. Take two close-up photos of plants. The first one should be representative of the majority of the plants in the field. The second one should be representative of a minority of plants in the field. The latter close-up photo should be captured only when the percentage of the minority class, in terms of area, is deemed significant (Fig. 1).

Fig. 1 helps to further illuminate what is meant by the terms primary, secondary and percentage of prevalence. First, it should be noted that the expert could only choose among the six principal phenological stages defined earlier in this section. The figure shows an example of two consecutive visits in a field. On the 5th of September the field was between the late BD phase and the early BO phase, with the majority of the plants having merely a couple of bolls opened.

Fig 1. Examples of field photos. Field photos that were captured in two consecutive visits of the same field. For each visit there is a panoramic photo of the field and two close-up photos of a plant that represent the majority and minority of the field, respectively. **For 05/09**, the primary stage is BD and the secondary stage is BO. **For 17/09**, primary is BO and secondary is BD.

The close-up photo labelled ”majority” shows a representative plant of this aggregate status of the the field, also conveyed by the panoramic photo, which can be described with BD as primary and BO as secondary. On the other hand, the close up photo labelled ”minority” refers to a plant that is less common in the field and is more representative of the secondary stage. This becomes even more clear by inspecting the ”minority” close-up photo for the 05/09 visit, which shows a plant that is well into the BO phase. The expert would identify the percentage of prevalence based on the area that the plants represented by the ”majority” and ”minority” close-up photos cover in the field, but also the average density of the two stages in each plant. In this example and for the 05/09 visit, BD was 100% and BO was 80% prevalent, which means that BD was found in every plant of the field while BO on 80% of the plants. Respectively, for the 17/09 visit BO was 100% and BD was 30% prevalent.

During the growing season of 2021, our expert made 1285 visits to 80 cotton fields in Orchomenos. Orchomenos is an agrarian municipality in Viotia district of central Greece. The fields that participated in the ground observation campaign are part of the Orchomenos cooperative that has the highest selling price for cotton in Greece. It should be noted that among the 80 fields, 10 different cotton varieties were cultivated. This variability is important, as one can evaluate the performance of the phenology estimation models and draw conclusions on their generalization. The field visits were appropriately scheduled in order to have minimal differences between ground and satellite observations. The mean difference between the ground and the Sentinel-2 observation was 0.67 days and the standard deviation was 0.58 days.

Table 1 shows the number of primary and secondary ground observations for each principal phenological stage. The expert was instructed to assign a primary stage label to any visit, thus the number of primary observations equals to the number of field visits. On the other hand, a secondary stage is not necessarily present. Specifically, a secondary stage was observed only in 669 out of the 1285 visits (52%).

Fig. 2 and Fig. 3 show the Kernel Density Estimation (KDE) of the Days of Year (DoY) for which the inspector observed the different principal phenological stages as primary and secondary, respectively. It becomes clear that there are many chronological overlaps among the stages, for both the primary and secondary annotations. Inspecting Fig. 2, we see that the overlaps get progressively larger as we move towards the end of the growing cycle. This is expected as differences in growth accumulate with time and thus get more pronounced. The two figures also highlight how different the rate of

Table 1. Distribution of phenological stages.

	Ground observations	
**Stage**	**Primary stage[a]**	**Secodary stage[b]**
**RE**	75	4
**LD**	421	20
**S**	212	5
**F**	229	148
**BD**	252	315
**BO**	96	177
**Total**	**1285**	**669**

The number of ground observations for each principal phenological stage of cotton that have been classified as [a]primary and [b]secondary stage labels.

growth can be even for fields that cultivate the same crop type, have similar sowing date and are in close proximity. Finally, it should be noted that for the secondary stage observations, the KDEs appear to have two modes (Fig. 3). The first and second mode refer to observations that were made prior and after the onset of the ”primary” phase of a phenological category. This is the reason why there are extensive overlaps among the KDEs for the secondary stage observations.

Fig 2. Distribution of DoY for which the inspector observed the phenological stages as primary.

Fig 3. Distribution of DoY for which the inspector observed the phenological stages as secondary.

The ground observation dataset is public (`https://github.com/Agri-Hub/cotton-phenology-dataset`), encouraging the community to use it for training, testing and evaluating the performance of cotton phenology estimation or yield estimation models. The dataset is given in the form of geojson and csv, including a) the geographic location and geometry of fields, b) the days of inspection, c) the primary phenological stage and the percentage it describes, d) the secondary phenological stage and the percentage it describes, e) the sowing and harvest dates, e) a panoramic photo of the field, f) close-up photos of representative plants for the ”majority” and ”minority” phenological stages.

The quality of the dataset has been evaluated by another expert, Expert 2, who reviewed a randomly selected subset of 145 ground observations using the available panoramic and close-up photos. Expert 2 was not aware of the ground observations and was asked to decide on the primary stage and secondary stage, if there was one. Then a third expert reviewed the disagreements between the decisions of Expert 1 and Expert 2 using once more the photos captured during the visits. Table 2 shows a number of metrics for the interrater agreement.

The analysis from Expert 3 yielded the percentages of direct agreement and disagreement, indirect agreement, N/A observations and undecided observations. N/A observations are the secondary stage observations that were considered unfair to include in the evaluation. In particular, we do not take into account the cases when Expert 1, i.e., the one that visited the field, provides only a primary stage observation and Expert 2 provides the same observation as a secondary stage. Such mismatch should be penalized only for the primary stage. The undecided category refers to instances that

Expert 2 claimed and Expert 3 confirmed that the photos were not good enough to make a fair assessment. The direct agreement refers to the percentages for which Expert 1 provided the exact same label in the correct order. The indirect agreement refers to the percentage for which the primary or secondary stage observations between Expert 1 and Expert 2 disagreed when the labels were viewed as annotations in an ordinal scale, but agreed when they were viewed as annotations in an interval scale. For this, Expert 3 used the percentages of prevalence provided by Expert 1. This becomes more clear with the following two examples:
Example 1: Expert 1 observes BD as primary at 100% and stage F as secondary at 60%, whereas Expert 2 observes BD as primary and none as secondary. Given that Expert 2 did not give percentages for the stages and that the predictions were based on just a few photos, the decisions are considered equivalent. In other words, there is direct agreement for the primary stage observation and indirect agreement for the secondary stage.
Example 2: Expert 1 observes F as primary at 100% and stage BD as secondary at 80%, whereas Expert 2 observes BD as primary and F as secondary. In this example, there is indirect agreement for both primary and secondary stages.

Table 2. Interrater agreement metrics[a].

	Primary stage	Secondary stage
**Direct agreement**	0.72	0.60
**Indirect agreement**	0.17	0.11
**Disagreement**	0.10	0.09
**N/A**	-	0.08
**Undecided**	0.01	0.11
**Krippendorff's alpha (ordinal)**	0.95	

[a] For the primary and secondary growth stage annotations (Expert 1 v. Expert 2).

Crop growth labeling is not straightforward because one attempts to derive ordinal categories to what is actually a continuous scale problem. Thus, the results will be heavily dependent on the choice of the category limits. Even more, those limits, although pre-defined and explained in detail to the various experts, are subject to different interpretations. The annotations collected through the field inspections are ordinal and thus we need to select an appropriate measure to reveal information on the reliability. Krippendorff's alpha is a versatile interrater agreement metric that is applicable to any level of meas[illegible]ment, e.g. nominal, ordinal or interval. The Krippendorff's alpha between Expert 2 and Experts 1 for ordinal level of measurement was 0.95. This indicates a strong agreement on the primary stage observations, which combined with the analysis performed by Expert 3, constitutes the annotation method reliable. From this point on, we only use Expert 1's ground observations. The aforementioned analysis was merely performed to assure the quality of the ground observations.

Predictor variables

Table 3 lists the predictor variable candidates with which we experimented in this study: i) the Sentinel-2 products and ii) the atmospheric and soil numerical simulations. In this section we focus on the acquisition and pre-processing the various prediction variables, whereas in the next sections we elaborate on how these variables are incorporated in the feature space and feed the phenology estimation models.

Table 3. Summary of predictor variable candidates.

Variable	Formula[a]	Resolution
Day of Year (DoY)	sine, cosine	-
Temperature at surface	min, max	2 km
Growing Degree Days (GDD) 2m	$(T_{max}-T_{min})/2 - T_{base}$	2 km
Accumulated Precipitation	max	2 km
Downwards Shortwave Radiation	max	2 km
Soil temperature 0-10 cm depth	min, max	2 km
Soil moisture 0-10 cm depth	min, max	2 km
Normalized Difference Vegetation Index (NDVI)	(B08-B04)/(B08+B04)	10 m
Normalized Difference Water Index (NDWI)	(B03-B08)/(B08+B03)	10 m
Normalized Difference Moisture Index (NDMI)	(B08-B11)/(B08+B11)	20 m
Plant Senescence Reflectance Index (PSRI)	(B04-B02)/ B06	20 m
Soil-Adjusted Vegetation Index (SAVI)	((B08 - B04)/(B08 + B04 + 0.428))*(1.0 + 0.428)	10 m
Enhanced Vegetation Index (EVI)	2.5*(B08-B04)/((B08+6*B04-7.5*B02) + 1.0)	10 m
Visible Atm. Resistant Indices Green (VARIgreen)	(B03-B04)/(B03+B04-B02)	10 m
Green Atmospherically Resistant Index (GARI)	(B08-(B03-(B02-B04)))/(B08-(B03+(B02-B04)))	10 m
Structure Insensitive Pigment Index (SIPI)	(B08-B02)/(B08-B04)	10 m
Wide Dynamic Range Vegetation Index (WDRVI)	(0.2*B08-B04)/(0.2*B08+B04)	10 m
Global Vegetation Moisture Index (GVMI)	((B08+0.1)-(B12 + 0.02))/((B08+0.1)+(B12+0.02))	20 m

[a] B is the spectral reflectance value of the band number of the Sentinel-2 image.

Optical images

The optical spectrum variables used in this study were derived from Sentinel-2 images. ~~These variables function as our eyes on the ground and enable the multi-temporal monitoring of crop status and development.~~ As mentioned earlier, optical SITS have been popular in related research studies, with special attention given to Sentinel-2, but also MODIS data. In some studies these two sources of optical images are used together, leveraging the high spatial resolution of Sentinel-2 data, and the high temporal resolution and thermal infrared bands of the MODIS sensor. The method of this work exploits only Sentinel-2 images in order to provide crop-specific phenology predictions at the field level. There are cases where the agricultural landscape is dominated by a single crop cultivation, e.g. U.S. Corn belt, and MODIS can be a tremendous help in crop-specific phenology predictions. But in Greece the landscape is fragmented and the medium spatial resolution of MODIS would yield mixed optical signatures of multiple crop types.

The optical component of this study's variable space comprises the RGB, NIR and SWIR spectral bands of Sentinel-2, but also a number of VIs. VIs are combinations of the spectral bands that can highlight particular vegetation properties [43]. Table 3 lists the various VIs that have been used in this work. The VIs investigated are some of the most common in the relevant literature. As the crop grows, its spectral signature changes with time. It starts with bare soil and then there are stems and leaves, and then flowers and bolls. These different phases have different biophysical and biochemical properties and thus different light reflectance profiles. Therefore we investigate multiple VIs so as to capture the maximum possible information at every stage of the growth. With regards to pre-processing, the Sentinel-2 images have been atmospherically corrected using the Sen2Cor software [44]. Additionally, clouds have been removed using the Sen2Cor scene classification product. Then, the null-valued pixels have been filled using linear interpolation on the SITS.

Atmospheric and soil parameters

A dense, long-term and efficient monitoring of the atmospheric state is rarely met in real-life conditions except for experimental campaigns. Automatic weather stations can contribute, but insufficient spatial coverage is a typical problem, let alone temporal gaps and discontinuities due to sensor failures or human aspects (poor maintenance). Given the absence of a dense in-situ network or weather radar scans over our area of study that could provide a necessary insight, we relied upon high resolution Numerical Weather Predictions (NWP) over Greece (2-km) from our convection-permitting operational configuration of WRF-ARW model [45]. The model is initialized daily with the latest available analysis and after the exclusion of the first few hours (spin-up time) in order to reach a statistical equilibrium, the following 24-hour estimates are utilized.

The specific parameters that were used in our pipeline were an outcome of consultation with agronomist experts and systematic literature review upon their correlation with the evolution of cotton. They include Air Temperature, Surface (skin) Temperature, 0-10cm Soil Temperature and Moisture, Precipitation and Incoming Shortwave Radiation. Growing Degree Days (GDD) is additionally computed, as it is one of the most essential indicators of phenology. Inspecting the GDD equation in Table 3, T_{max} and T_{min} are the maximum and minimum daily air temperatures (at 2 m) and T_{base} is the crop's base temperature (15.6 °C). The latter is defined as the temperature below which cotton does not develop. The GDD variable is also known as thermal time and is an indicator of the effective growing days of the crop [46].

Fuzzy clustering

We propose a fuzzy clustering method for the within-season estimation of cotton phenology. The workflow of the proposed approach is depicted in Fig. 4. We use clustering to circumvent the ever-present problem of sparse, scarce and difficult to acquire ground observations, which constitute the supervised alternatives of limited applicability in operational scenarios. Nevertheless, we visited tens of fields and collected hundreds of ground observations in order to test the performance of our models. The aim of our newly introduced ground observation collection protocol was to extract more information than the principal growth stages, as it is usually the case in related works. This happens at the labelling level via taking advantage of the primary and secondary stage ground observations, as described in detail earlier.

Fig 4. The proposed methodology for cotton phenology estimation.

$\mathcal{L} = \{\lambda_1, \lambda_2, ..., \lambda_k\}$ is the finite ordered scale of the principal cotton growth class labels, where λ_1 is RE and λ_6 is BO. The ground observation protocol allowed k=6 phenological stages to choose from and a maximum of n = 2 labels to assign to each field, i.e., the primary and secondary stage categorization. This restricted the set of allowable labels L_r to all possible permutations of n = 2 elements of $\mathcal{L}$ and all unit sets. Specifically, there are $k^2 = 36$ allowable labels. This multi-label problem can be reduced to a single-label one by considering each subset as a distinct metaclass [47]. In reality, however, there are only 16 possible metaclasses, as the primary and secondary stage for a field can differ only a single position in the ordinal scale. The ~~following~~ equation lists this set of 16 metaclasses in ordered scale.

$$L_r = \{\lambda_1, (\lambda_1, \lambda_2), (\lambda_2, \lambda_1), \lambda_2, (\lambda_2, \lambda_3), (\lambda_3, \lambda_2), \lambda_3, (\lambda_3, \lambda_4), (\lambda_4, \lambda_3), \lambda_4, (\lambda_4, \lambda_5), (\lambda_5, \lambda_4), \lambda_5, (\lambda_5, \lambda_6), (\lambda_6, \lambda_5), \lambda_6\} \quad (1)$$

In order to estimate the phenological metaclass for each field at any given instance, we used an FCM clustering algorithm. $X \in R^{K \times E}$ denotes the element space that was used as input to the FCM, where K is equal to the number of fields (M) multiplied by the number of Sentinel-2 acquisitions (J), and E is equal to the number of features. Each row of the two-dimensional space in Eq 2 represents field i at the time instance j (DoY of Sentinel-2 acquisition). For the Sentinel-2 variables, each element $x_{(i,j),d}$ gets the mean value of variable d for all pixels of field i at the instance j. In the same fashion, for the NWP variables, each element $x_{(i,j),d}$ gets the value of the nearest grid cell to field i for the instance j. To calculate the mean value of the pixels and grid cells that fall within each field we used the parcel boundaries retrieved from the Hellenic Cadastre.

$$X_{(i,j),d} = \begin{pmatrix} x_{(1,1),1} & x_{(1,1),2} & \cdots & x_{(1,1),E} \\ x_{(1,2),1} & x_{(1,2),2} & \cdots & x_{(1,2),E} \\ \vdots & \vdots & \ddots & \vdots \\ x_{(1,J),1} & x_{(1,J),2} & \cdots & x_{(1,J),E} \\ \vdots & \vdots & \ddots & \vdots \\ x_{(M,J),1} & x_{(M,J),2} & \cdots & x_{(M,J),E} \end{pmatrix} \tag{2}$$

Since phenology is dependent on the relative temporal progression of variables, we use the accumulated NWP parameters and the cumulative integrals of the VIs. The starting point for the accumulation is set around the earliest sowing DoY for the fields in the area of interest. In our case, this starting point was the 10th of April (DoY 100). Therefore, the feature space includes the Sentinel-2 VIs and their cumulative integrals, the accumulated NWP parameters, and the cosine and sine of the Sentinel-2 acquisition DoY (Table 3).

During the learning phase, the FCM algorithm attempts to partition K elements $X = \{\boldsymbol{x}_1, ..., \boldsymbol{x}_K\}$ that capture the entirety of the season into c = 6 clusters that is assumed they represent the six principal growth stages of cotton (after-season clustering in Fig. 4) [48]. This is considered to be a valid assumption given that each element is described by the EO and NWP variables, their time-accumulated variants, and the associated DoY. The assumption is also supported by the results in the next section. The algorithm returns a list of $C = \{\boldsymbol{c}_1, \boldsymbol{c}_2, ..., \boldsymbol{c}_6\}$ cluster centers and a partition matrix $W = (w_{k,l}) \in R^{K \times c}$, where $w_{k,l}$ is the degree to which the element $\boldsymbol{x}_k$ belongs to cluster $\boldsymbol{c}_l$. We applied FCM on the 2020 variable space (training year) in Orchomenos and then used the clusters (C) to produce within-season predictions, in dynamic fashion, during the 2021 season (test year). The training cotton fields of 2020 were 194 in total, and were extracted from a pre-trained crop classification model, based on [49].

After the clustering, the phenological stages are assigned to the different clusters via exploiting the time order. For this, the most common order of clusters is recorded and then matched to the ordered scale of labels in $\mathcal{L}$. It is common to address a multi-label problem in an indirect way using a scoring function $f : X \times \mathcal{L} \rightarrow R$ that assigns a real number to each element-label pair [47]. The assumption here is that this scoring function corresponds to the probability of each label being relevant to an element. In our case, the scoring function f is the FCM and the scores are the membership grades $w_{k,j}$ of each element $\boldsymbol{x}$. In other words, the FCM attempts to find the labels in $\mathcal{L}$ and then the partition scores or weights are used for multi-label prediction via thresholing. Even more, sorting the labels according to their score provides label ranking, enabling the identification of the primary and secondary stages, as given through the field inspections (Eq 3).

$$\lambda_i \leq_x \lambda_j \Leftrightarrow f(\boldsymbol{x}, \lambda_i) \leq f(\boldsymbol{x}, \lambda_j) \tag{3}$$

Evaluation metrics

We considered the machine learning task of phenology estimation as a multi-label classification problem, given the potential duality of phenological stages at a given instance. The two labels are ranked as primary and secondary phenological stages according to their relevance, or prevalence, in the field. Therefore, the metrics for assessing our model should capture these properties.

First, we categorize the predictions to error classes according to the difference or displacement between the prediction and the ground observation in the ordinal scale of metaclasses. These error classes are labelled as diff-o, with $o \in \{0, 1, 2, 3\}$. For instance, if our model predicted λ_2 and the ground observation was (λ_3, λ_2), then according to Eq 1 the prediction is categorized as diff-2. Similarly to the Top-N accuracy, we devised the maxdiff-o accuracy, with $o \in \{0, 1, 2, 3\}$, measuring the percentage of predictions with a displacement no greater than o. For instance, maxdiff-2 is the percentage of predictions that have at most an error of two displacement units. We also use the well known kappa coefficient, together with its linear and quadratic weighted variants. The weighted kappa metrics allow for disagreements to be weighted differently and are commonly used when labels are ordered.

We additionally incorporate the Normalized Discounted Cumulative Gain (NDCG). It is a popular metric in the world of information retrieval and specifically in tasks such as top-N ranking and item recommendations. In our case, we use the various partition weights or membership probabilities $(w_{k,j})$ as relevance values and rank the phenological clusters accordingly. We use NDCG@2, as we take into account only the top 2 ranked stages/clusters that we assume represent the primary and secondary annotations. The highly relevant phenology stage is better to be ranked higher than the less relevant stage, which is in turn better to be ranked higher than non-relevant stages. NDCG@2 captures and evaluates exactly this capability of the model.

NDCG is based on the cumulative gain that simply sums the relevance scores for top@p $(p = 2)$. This is mathematically expressed by:

$$CG_p = \sum_{i=1}^{p} rel_i \tag{4}$$

In this case, we set $rel = 2$ for the primary stage and $rel = 1$ for the secondary stage. The cumulative gain however does not take into account the position of the phenological stage in the rank. This is done by the discounted cumulative gain, as in Eq 5, which makes use of a log-based penalty function and reduces the relevance score that is normalized by a penalty equivalent to each position.

$$DCG_p = \sum_{i=1}^{p} \frac{rel_i}{\log_2(i+1)} = rel_1 + \sum_{i=2}^{p} \frac{rel_i}{\log_2(i+1)} \tag{5}$$

Finally, the discounted cumulative gain is simply normalized by the ideal order of the relevant items and we end up with NDCG [50].

Results

The FCM clustering was performed on the EO and NWP variables for the Orchomenos region in 2020 (training year) and was then applied on the equivalent feature space of 2021 (test year) for the within-season prediction of phenological metaclasses. Our FCM-based approach has three parameters, i) the number of clusters, ii) the fuzzifier $m \in R$, with $m \geq 1$ and iii) the partition score threshold, above which a cluster is considered as a secondary stage label. The number of clusters was fixed at $c = 6$ in

order to force the FCM to discover all six principal phenological stages. The fuzzifier m was set to 2, which is commonly preferred when using the FCM algorithm [51–54]. According to [55] the best choice for m is in the interval [1.5, 2.5], with $m = 2$ being the most common choice. Finally, the partition threshold ($\mathrm{th_w}$) depends on the distribution of the partition scores during the learning phase of the FCM algorithm.

For each element $\boldsymbol{x}$, FCM gives a partition weight $\mathrm{w_{k,j}}$ that refers to the degree the element belongs to each of the clusters. The weights are then sorted for each element. The two-labelled nature of our target (primary and secondary stages) implies that the values of partition weights ranked 3rd or lower, should not be considered as valid phenological stage labels. Thus, we set the threshold $\mathrm{th_w}$ equal to the value of the 98th percentile of the partition weights ranked in the third place (Fig. 5). We used the 98th percentile to eliminate the influence of potential outliers. Indicatively, Fig. 5 illustrates the distribution of the partition weights (for $m = 2$) for a representative model. The different colors indicate the rank in which the weights were given for each prediction, i.e., from 1st to 6th. In this case, the threshold is set to 0.11 based on the aforementioned rule. A different threshold was computed for each different model that we tested.

Fig 5. Distribution of FCM partition weights for m=2. The hue indicates the rank in which the weights were given for each prediction. The dotted line shows the threshold above which 2nd ranked clusters are considered as valid secondary phenological stages.

Having configured the three parameters, we trained FCM models (after-season prediction) on the 2020 cotton fields (training year), with multiple combinations of features. The FCM algorithm works well in low dimensions. Specifically, [56] suggested that for dimensions larger than ten, the FCM starts to present ill behaviour; and for this reason we ran our experiments on hyperspaces of up to ten features. We had 32 features to choose from, i.e., the variables described in Table 3 and their cumulative variants, and generated feature sets of length 3 to 10 features. However, an exhaustive analysis would have required more than 200 million clusterings. To avoid running so many experiments, we fitted the FCM for feature sets of length $E \in \{3, ..., 10\}$ using ten thousand random feature combinations for each feature set size. It should be noted that all feature sets include the features cos(DoY) and sin(DoY) that set the time frame.

In order to evaluate the performance of the algorithm on the different feature spaces, we applied each individual FCM model (i.e., the cluster centers, C, and thresholds, $\mathrm{th_w}$, from the training year) on the 2021 cotton fields, for which we have ground truth labels. We split the data into test and validation (70-30), and then used the kappa coefficient values of the validation data to find the best 1% (mean kappa = 0.45) of feature combinations. Based on these top feature combinations, we selected the 15 most appeared features. These were i) the VIs SIPI, GVMI, EVI, NDMI and SAVI, ii) the cumulative integrals of WRDVI, PSRI, NDWI and SIPI, iii) the accumulated maximum soil temperature, maximum surface temperature, maximum solar radiation and Accumulated GDD (AGDD) and iv) the always present time features of sin(DoY) and cos(DoY).

Having concluded to the aforementioned set of predictors, we ran the FCM algorithm again for the training year (2020), for every possible combination of those features in spaces of length 6 to 15. This time, apart from the kappa coefficient, we also considered the maxdiff-1 score. Specifically, we kept solutions with kappa coefficient larger than 0.46 and maxdiff-1 larger than 0.86, which resulted to 604 cases out of 7,814. These values were set so, in order to acquire better solutions compared to a baseline model. The baseline model refers to an FCM with only DoYs as input. Since phenology is closely related to the DoY, the baseline is used to capture this chance agreement and showcase by comparison the real competence of our model. Detailed comparisons with

the baseline model follow later in this section.

The analysis revealed that for most cases feature sets of length $E > 10$ yielded sub-optimal results. Specifically, from the top 604 models, 84% contained no more than 10 features. Besides the DoY features, the AGDD is by far the most common feature since it appeared in more than 86% of the best solutions. Another important observation here is that the cumulative integrals of VIs and the accumulated NWP features are more important than than the single-date VIs. The importance of these features is great since they capture the dimension of time, which is essential for an unsupervised phenology prediction model. Nevertheless, the results showed that the majority of well-performing feature sets contained at least one feature from each category, namely VIs, cumulative integrals of VIs and accumulated NWPs.

From the top 15 features, the cumulative integral of SIPI, the accumulated maximum solar radiation, NDMI and SIPI did not appear as frequently in the best 604 models. For this reason, we discarded models that included these features. The final set of models, which was used for our predictions, comprised models with 8 and 9 features that included at least one feature from each category. This resulted to a total of 82 models. In order to ensure the generalization and robustness of our methodology, by not depending on a single feature set, we generate the final predictions through majority voting on those best models. The 82 feature sets are listed in S1 Table.

Table 4 shows the performance of our model and the baseline model on the test set. Indeed, it is shown that our model provides a significantly larger number of diff-0 predictions, namely perfect agreements between predicted and ground truth metaclasses. This is also evident via observing Cohen's kappa that is notably higher for our model. It is also worth mentioning how we largely outperform the baseline in terms of NDCG, denoting a better ranking capacity.

Table 4. Metrics of performance for our phenology prediction model and the baseline model.

	Ours	Baseline
**maxdiff-0**	0.53	0.38
**maxdiff-1**	0.88	0.86
**maxdiff-2**	1.00	0.97
**maxdiff-3**	1.00	1.00
**Cohen's kappa**	0.48	0.33
**Weighted kappa (Linear)**	0.88	0.84
**Weighted kappa (Quadratic)**	0.98	0.97
**NDCG**	0.93	0.88

Table 5 shows the prediction errors in metaclass displacement units, for phenology metaclasses that had at least 10 ground observations. The displacement units are computed via multiplying the normalized confusion matrix with a weight matrix, for which the cells one off the diagonal of the confusion matrix are weighted 1, those two off are weighted 2, etc. Then the weighted displacement units are summed for each ground observation metaclass. Our model offers a smaller average displacement for six out of the eight metaclasses that account for the majority of ground observations.

It is observed that the metaclass (BO, BD) offers the smallest error in displacement units, whereas the (F, BD) metaclass gives by far the largest. This can be explained by the fact that that metaclass (BO, BD) is at the edge of the growing cycle and can only be confused with the metaclass that precedes it. On the other hand, metaclass (F, BD) is at the vegetation peak, where plants are well into the flowering phase and some bolls have started to develop. The consecutive metaclasses (F, -), (F, BD) and (BD, F) are situated near the plateau that is formed around the peak of the VI time-series curve (or

Table 5. Prediction errors in metaclass displacement units.

Metaclass		Support	Displacement	
			Ours	Baseline
1	(RE, -)	72	**0.41**	1.33
4	(LD, -)	415	**0.62**	0.89
6	(S, LD)	17	**0.41**	1.00
7	(S, -)	122	0.58	**0.17**
8	(S, F)	73	**0.30**	0.56
11	(F, BD)	225	1.04	**0.92**
12	(BD, F)	75	**0.37**	0.75
14	(BD, BO)	177	**0.54**	0.72
15	(BO, BD)	90	**0.03**	0.68
**Average**		1266	**0.48**	0.78

valley, given the VI). Therefore, there are not significant differences in VI values among the three metaclasses, which explains the less than optimal performance for metaclass (F, BD). Overall, the average displacement shows significant difference between the two models. Our model achieves a respectable average error of less than half a metaclass.

Fig. 6 shows the confusion matrix for the hard clustering predictions. It can be observed that for the principal phenological stages the model performs rather well, with an overall accuracy 87%. Most misclassifications are observed for the BD stage. This is actually expected given the number of observations for which BD is observed in one of the transitional metaclasses (Table 5). As a matter of fact, BD is never observed as unit set metaclass.

Fig 6. Confusion matrix for the six principal phenological stages.

As mentioned, the expert would visit each field three or four times a month. Thus, it was common to observe a field in a particular crop growth stage for multiple consecutive visits (3 to 6). The chronological order of observation, i.e., the relative position of the ground observation in the range enclosed between the first and last time a stage was observed as primary for a particular field, is useful since it indicates if it is observed in its early, middle or late phases.

Fig. 7 shows the distribution in the order of observation for the different disagreement categories, diff-o. The order of observation is categorized into early, middle and late visits. For a field that was observed in a particular phenological stage for three to five consecutive visits, early visit was the first visit and late visit was the last visit. In the seldom cases that a phenological stage was observed in six consecutive visits, then the first two and last two would be characterized as early and late visits, respectively.

The figure shows that the majority of the perfect agreements, diff-0, were mostly middle and late observations. On the other hand the vast majority of big disagreements, i.e., diff-1, diff-2 and diff-3, were for early stage observations or, for fewer cases, late stage observations. This is expected, as middle observations would indicate that the field is well into a particular stage, whereas early or late observations denote transitional phases.

Fig 7. Distribution of the chronological order of observation. Early describes ground observations that are found at the beginning of consecutive observations of a stage, with regards to chronological order. Late, similarly, describes ground observations that are found at the end of consecutive observations of a stage and Middle contains ground observations that are found in middle positions The hues show the predictions according to their diff-*o* categorization of prediction error.

Discussion

Land surface phenology

The results indicated that using clustering for the within-season phenology estimation is promising but challenging. EO and NWP data are competent predictor variables for this type of problems, as they represent both the land cover changes and the crop growth drivers, but cannot fully capture the physiological growth stages of crops. Furthermore, for operational applications in agricultural management, phenology predictions should be made at a within-season basis. However, there are still several challenges in this undertaking. First, within-season predictions are possible using only a part of the data time-series. Additionally, temporally dense EO time-series are crucial to ensure that critical phenological changes are detected as soon as possible [1]. The density of the time-series however is subject to certain trade-offs. Considering the freely available satellite images, higher temporal resolution implies lower spatial resolution. Furthermore, optical SITS is significantly affected by cloud coverage. This might have not proved a problem for this study, having its area of interest in Greece, but is certainly an issue for other parts of the world. Finally, crop growth stages might not be directly related to EO and NWP atmospheric and soil variables. For this, there is a clear gap in the literature for sophisticated modelling that would be able to capture these complex relationships.

Ground observations

Ground observations are required to assess how well the EO and NWP based land surface phenology relates with the actual phenological stages. Most validation datasets are focused only on few stages using aggregated statistics over large areas [1]. The National Agriculture Statistics Service (NASS) crop progress reports is such an example. Field-level ground observations are limited and rarely systematic. Recently, phenocams have been used to evaluate land surface phenology approaches [57]. However, the network of phenocams is still sparse and confined. Furthermore, labeling phenological stages is a complex process and cannot be fully solved by observing photos from the field. Therefore, ground observations are still a necessity.

In this regard, this study offers a unique dataset of ground observations at the field level. The ground observations are accompanied by a panoramic and a couple of close-up photos of representative plants. We introduce a new field campaign protocol for collecting ground observations for crop growth that allows up to two label assignments. If two labels are assigned then the inspector should specify which one is the primary growth stage and which one is the secondary growth stage that describes the field. This allows for the detailed description of crop growth stages through the metaclasses that result from combining the primary and secondary stage annotations. Additionally, not having to decide on a single label makes the ground observation easier and can potentially increase the number of people who can perform them. This is true since the choice of the limits that define the principal growth stages differs among studies and ground observation protocols. Even more, closer to the start and end of those limits it

gets tricky to decide on a single label. Having two labels can enable the reliable and large-scale crowdsourcing of ground observations.

Furthermore, the reliability of the ground observation collection method has been thoroughly evaluated. For this, we used the blinded interpretation of the field photos by an expert. Then the decisions of the field inspector and the photo inspector were evaluated by a third expert that decided on the direct agreement, indirect agreement and overall disagreement. The quality assurance process yielded satisfactory results, deeming the field campaign outputs reliable. The community is thus encouraged to use the openly available dataset and test their own models. The dataset is accompanied by the photos captured during the visits, which can be used for further interpretation but also computer vision tasks, such as crop classification and phenology classification.

Clustering for phenology estimation

It was shown that the introduced clustering method managed to learn from the complete time-series of 2020 and successfully infer the phenological metaclasses in a within-season fashion for 2021. Our model significantly outperforms the baseline, making the proposed approach very promising. Furthermore, the model predicts 16 different metaclasses and goes beyond the 6 principal phenological stages, extracting more information on their fuzzy transitions. This is particularly important since more intricate and precise agricultural management is now possible at the field level.

In many studies, phenology estimation is addressed as a regression problem, aiming to predict the DoYs of growth stage onsets, which is essential information for operational applications. This study's metaclass approach can also be viewed as a classification-based alternative of onset detection. The fuzzy metaclasses (λ_a, λ_b) and (λ_b, λ_a) denote this transitional phase between principal growth stages. In other words, the end of stage a and the onset of stage ¸ respectively. In future work, further testing will be conducted for the evaluation of the spatial and temporal generalization of the proposed methodology. This will require additional ground observations at different areas and years of inspection.

We showed that formulating phenology estimation as a clustering problem, via incorporating the time in our features, is a valid one. The authors suggest that there is great potential and encourage the community to test more models on the proposed premise. During experiments, it was observed that the FCM was sensitive on the DoY of the first and last element included in the learning phase. This is expected since the clustering is largely dependent on time component of our features. It is therefore important to set the "start" and "end" instances with the aim to enclose the average length of the cotton season. This is easy when the sowing and harvest dates are known, but could also be approximated via observing the mean and standard deviation of the VI time-series.

This work relied on i) feature engineering, incorporating the time in the form of cumulative EO and NWP variables, and ii) feature selection to decide on a set of optimal feature spaces. Tens of thousands of experiments were performed for feature selection, yielding robust results. The robustness lies in the fact that the top 15 features systematically appeared in the best performing models. Furthermore, phenology predictions are based on the majority vote of the best-performing combinations of the top features, making sure our approach can generalize.

Having said that, there is a number of recent studies that look into DL based unsupervised change detection on SITS [58–61]. We see great potential in such approaches and we believe could be applicable in the proposed unsupervised premise for phenology estimation. Common denominator of these methods is the learning of a smaller latent or embedding space, in which entities that bear resemblance are located closer to each other. This is particularly important for clustering techniques that aim to

group similar samples in the hyperspace. Usually, clustering algorithms, such as FCM, measure this similarity among entities using pair-wise distances. It is known that high dimensional spaces are not ideal for distance based techniques, as they usually fail to capture meaningful clusters. In addition, a latent manifold representation is not greatly dependent on feature engineering and can generalize well.

Conclusion

In this paper we proposed a fuzzy clustering method for the within-season phenology estimation for cotton in Greece. Our method is unsupervised to tackle the problem of sparse, scarce and hard to acquire ground observations. It provides predictions within-season and thus enables its usage in operational agricultural management scenarios. It focuses on cotton which is important for three reasons - i) it is an underrepresented crop type in related literature, ii) the relationship between remote sensing phenology and the physiological growth of cotton is complex and iii) cotton is a very important crop for the economy and agricultural ecosystem of Greece, which is the study area.

We conducted field visits to collect ground observations that are offered to the community as a ready-to-use label dataset. For this, we used a new protocol that leverages two ranked labels. This makes the observations easier and at the same time provides enhanced information on the growth status. Therefore, we approach the problem as a multi-label one, introducing the notion of metaclasses. We go beyond the principal phenological stages of cotton by providing prediction for 16 metaclasses using the membership probabilities of the FCM classifier.

Finally, we experimented with numerous combinations of features, including accumulated numerical simulations of atmospheric and soil paramaters, Sentinel-2 based VIs and their cumulative integral variants. Based on these experiments, we provided a list of optimal feature sets that can be used for cotton phenology estimation through majority voting.

Supporting information

S1 Table. The feature sets of the top 82 FCM models with size 8 or 9 features. With (I) we show the cumulative integrals of the VIs. max_soil refers to the cumulative maximum soil temperature, max_surf to the cumulative maximum surface temperature and wkappa to the linear weighted kappa coefficient. The last five columns refer to the performance metrics.

Acknowledgments

The authors express their gratitude to Mr. Vaggelis Dedes for performing the ground observation campaign and Dr. Dimitra A. Loka, Researcher at the Institute of Industrial and Forage Crops in Greece, for her valuable consultations. Finally, the authors acknowledge the farmers' association of Orchomenos (ASOO) for letting us perform the ground observations in their fields and for giving consent to release these in a publicly available dataset.

References

1. Gao F, Zhang X. Mapping crop phenology in near real-time using satellite remote sensing: Challenges and opportunities. Journal of Remote Sensing. 2021;2021.

2. Vellidis G, Liakos V, Andreis J, Perry C, Porter W, Barnes E, et al. Development and assessment of a smartphone application for irrigation scheduling in cotton. Computers and Electronics in Agriculture. 2016;127:249–259.

3. Oosterhuis DM, Bourland FM. In: Physiological Cutout. Agricultural Experiment Station Fayetteville: University of Arkansas, Division of Agriculture; 2008.Available from: `http://www.cotman.org/files/COTMAN-Manual.pdf`.

4. Anderson MC, Hain CR, Jurecka F, Trnka M, Hlavinka P, Dulaney W, et al. Relationships between the evaporative stress index and winter wheat and spring barley yield anomalies in the Czech Republic. Climate Research. 2016;70(2-3):215–230.

5. Tang J, Körner C, Muraoka H, Piao S, Shen M, Thackeray SJ, et al. Emerging opportunities and challenges in phenology: a review. Ecosphere. 2016;7(8):e01436. doi:https://doi.org/10.1002/ecs2.1436.

6. Sitokonstantinou V, Koutroumpas A, Drivas T, Koukos A, Karathanassi V, Kontoes H, et al. A Sentinel based agriculture monitoring scheme for the control of the CAP and food security. In: Eighth International Conference on Remote Sensing and Geoinformation of the Environment (RSCy2020). vol. 11524. International Society for Optics and Photonics; 2020. p. 1152407.

7. Lacueva-Pérez FJ, Ilarri S, Vargas JJB, Lezaun GL, del Hoyo-Alonso R. Multifactorial Evolutionary Prediction of Phenology and Pests: Can Machine Learning Help? In: WEBIST; 2020. p. 75–82.

8. Potgieter AB, Zhao Y, Zarco-Tejada PJ, Chenu K, Zhang Y, Porker K, et al. Evolution and application of digital technologies to predict crop type and crop phenology in agriculture. in silico Plants. 2021;3(1):diab017.

9. Liang L, Schwartz MD, Fei S. Validating satellite phenology through intensive ground observation and landscape scaling in a mixed seasonal forest. Remote Sensing of Environment. 2011;115(1):143–157.

10. Xin Q, Li J, Li Z, Li Y, Zhou X. Evaluations and comparisons of rule-based and machine-learning-based methods to retrieve satellite-based vegetation phenology using MODIS and USA National Phenology Network data. International Journal of Applied Earth Observation and Geoinformation. 2020;93:102189.

11. Almeida J, Dos Santos JA, Alberton B, Torres RdS, Morellato LPC. Remote phenology: Applying machine learning to detect phenological patterns in a cerrado savanna. In: 2012 IEEE 8th International Conference on E-Science. IEEE; 2012. p. 1–8.

12. Verbesselt J, Hyndman R, Zeileis A, Culvenor D. Phenological change detection while accounting for abrupt and gradual trends in satellite image time series. Remote Sensing of Environment. 2010;114(12):2970–2980.

13. Tian J, Wang L, Yin D, Li X, Diao C, Gong H, et al. Development of spectral-phenological features for deep learning to understand Spartina alterniflora invasion. Remote Sensing of Environment. 2020;242:111745.

14. Bolton DK, Gray JM, Melaas EK, Moon M, Eklundh L, Friedl MA. Continental-scale land surface phenology from harmonized Landsat 8 and Sentinel-2 imagery. Remote Sensing of Environment. 2020;240:111685.

15. Dineshkumar C, Nitheshnirmal S, Bhardwaj A, Priyadarshini KN. Phenological monitoring of paddy crop using time series modis data. In: Multidisciplinary Digital Publishing Institute Proceedings. vol. 24; 2019. p. 19.

16. Chen J, Rao Y, Shen M, Wang C, Zhou Y, Ma L, et al. A simple method for detecting phenological change from time series of vegetation index. IEEE Transactions on Geoscience and Remote Sensing. 2016;54(6):3436–3449.

17. Lopez-Sanchez JM, Vicente-Guijalba F, Ballester-Berman JD, Cloude SR. Estimating phenology of agricultural crops from space. In: ESA Living Planet Symp., Edinburgh, UK; 2013.

18. Yang Z, Shao Y, Li K, Liu Q, Liu L, Brisco B. An improved scheme for rice phenology estimation based on time-series multispectral HJ-1A/B and polarimetric RADARSAT-2 data. Remote sensing of environment. 2017;195:184–201.

19. Nieto L, Schwalbert R, Prasad P, Olson BJ, Ciampitti IA. An integrated approach of field, weather, and satellite data for monitoring maize phenology. Scientific Reports. 2021;11(1).

20. Zheng H, Cheng T, Yao X, Deng X, Tian Y, Cao W, et al. Detection of rice phenology through time series analysis of ground-based spectral index data. Field Crops Research. 2016;198:131–139.

21. Czernecki B, Nowosad J, Jabłońska K. Machine learning modeling of plant phenology based on coupling satellite and gridded meteorological dataset. International journal of biometeorology. 2018;62(7):1297–1309.

22. Vicente-Guijalba F, Martinez-Marin T, Lopez-Sanchez JM. Crop phenology estimation using a multitemporal model and a Kalman filtering strategy. IEEE Geoscience and Remote Sensing Letters. 2013;11(6):1081–1085.

23. De Bernardis C, Vicente-Guijalba F, Martinez-Marin T, Lopez-Sanchez JM. Particle filter approach for real-time estimation of crop phenological states using time series of NDVI images. Remote Sensing. 2016;8(7):610.

24. Sakamoto T, Wardlow BD, Gitelson AA, Verma SB, Suyker AE, Arkebauer TJ. A two-step filtering approach for detecting maize and soybean phenology with time-series MODIS data. Remote Sensing of Environment. 2010;114(10):2146–2159.

25. Lausch A, Salbach C, Schmidt A, Doktor D, Merbach I, Pause M. Deriving phenology of barley with imaging hyperspectral remote sensing. Ecological Modelling. 2015;295:123–135.

26. Zeng L, Wardlow BD, Wang R, Shan J, Tadesse T, Hayes MJ, et al. A hybrid approach for detecting corn and soybean phenology with time-series MODIS data. Remote Sensing of Environment. 2016;181:237–250.

27. Tsiros E, Domenikiotis C, Dalezios N. Assessment of cotton phenological stages using agroclimatic indices: An innovative approach. Italian Journal of Agrometeorology. 2009; p. 50–55.

28. Reddy K, Hodges HF, McKinion JM. A temperature model for cotton phenology. Biotronics. 1993;22.

29. Reddy V. Modeling cotton growth and phenology in response to temperature. Computers and electronics in agriculture. 1994;10(1):63–73.

30. Cai Y, Guan K, Lobell D, Potgieter AB, Wang S, Peng J, et al. Integrating satellite and climate data to predict wheat yield in Australia using machine learning approaches. Agricultural and forest meteorology. 2019;274:144–159.

31. Meroni M, d'Andrimont R, Vrieling A, Fasbender D, Lemoine G, Rembold F, et al. Comparing land surface phenology of major European crops as derived from SAR and multispectral data of Sentinel-1 and-2. Remote sensing of environment. 2021;253:112232.

32. Arun PV, Karnieli A. Deep Learning-Based Phenological Event Modeling for Classification of Crops. Remote Sensing. 2021;13(13):2477.

33. Zhang X, Friedl MA, Schaaf CB, Strahler AH, Hodges JC, Gao F, et al. Monitoring vegetation phenology using MODIS. Remote sensing of environment. 2003;84(3):471–475.

34. Vina A, Gitelson A, Rundquist D, Keydan G, Leavitt B, Schepers Js. Monitoring maize (Zea mays L.) phenology with remote sensing. Agronomy Journal. 2004;96.

35. Yang Q, Shi L, Han J, Yu J, Huang K. A near real-time deep learning approach for detecting rice phenology based on UAV images. Agricultural and Forest Meteorology. 2020;287:107938.

36. Selvaraj MG, Valderrama M, Guzman D, Valencia M, Ruiz H, Acharjee A. Machine learning for high-throughput field phenotyping and image processing provides insight into the association of above and below-ground traits in cassava (Manihot esculenta Crantz). Plant methods. 2020;16(87).

37. Wang XA, Tang J, Whitty M. DeepPhenology: Estimation of apple flower phenology distributions based on deep learning. Computers and Electronics in Agriculture. 2021;185:106123.

38. Sitokonstantinou V, Koukos A, Kontoes C, Bartsotas NS, Karathanassi V. Semi-Supervised Phenology Estimation in Cotton Parcels with Sentinel-2 Time-Series. In: 2021 IEEE International Geoscience and Remote Sensing Symposium IGARSS; 2021. p. 8491–8494.

39. Sakamoto T, Yokozawa M, Toritani H, Shibayama M, Ishitsuka N, Ohno H. A crop phenology detection method using time-series MODIS data. Remote sensing of environment. 2005;96(3-4):366–374.

40. COTTON EVALUATION MANUAL; 2003. Available from: `https://www.elga.gr/images/stories/egxeiridia/VAMVAKI.pdf`.

41. Danalatos N. An introduction in crop production simulation; 2007.

42. Meier U. Growth stages of mono-and dicotyledonous plants. Blackwell Wissenschafts-Verlag; 1997.

43. Segarra J, Buchaillot ML, Araus JL, Kefauver SC. Remote sensing for precision agriculture: Sentinel-2 improved features and applications. Agronomy. 2020;10(5):641.

44. Louis J, Debaecker V, Pflug B, Main-Knorn M, Bieniarz J, Mueller-Wilm U, et al. SENTINEL-2 SEN2COR: L2A Processor for Users. In: Ouwehand L, editor. ESA Living Planet Symposium 2016. vol. SP-740 of ESA Special Publications (on CD). Spacebooks Online; 2016. p. 1–8. Available from: https://elib.dlr.de/107381/.

45. Skamarock WC, Klemp JB, Dudhia J, Gill DO, Liu Z, Berner J, et al. A description of the advanced research WRF model version 4. National Center for Atmospheric Research: Boulder, CO, USA. 2019; p. 145.

46. Sharma A, Deepa R, Sankar S, Pryor M, Stewart B, Johnson E, et al. Use of growing degree indicator for developing adaptive responses: A case study of cotton in Florida. Ecological Indicators. 2021;124:107383.

47. Cheng W, Dembczynski K, Hüllermeier E. Graded Multilabel Classification: The Ordinal Case. In: ICML; 2010.

48. Bezdek JC, Ehrlich R, Full W. FCM: The fuzzy c-means clustering algorithm. Computers & geosciences. 1984;10(2-3):191–203.

49. Sitokonstantinou V, Koukos A, Drivas T, Kontoes C, Papoutsis I, Karathanassi V. A Scalable Machine Learning Pipeline for Paddy Rice Classification Using Multi-Temporal Sentinel Data. Remote Sensing. 2021;13(9):1769. doi:https://doi.org/10.3390/rs13091769.

50. Järvelin K, Kekäläinen J. Cumulated gain-based evaluation of IR techniques. ACM Transactions on Information Systems (TOIS). 2002;20(4):422–446.

51. De Oliveira JV, Pedrycz W. Advances in fuzzy clustering and its applications. John Wiley & Sons; 2007.

52. Singh BK, Verma K, Thoke AS. Fuzzy cluster based neural network classifier for classifying breast tumors in ultrasound images. Expert Systems with Applications. 2016;66:114–123. doi:https://doi.org/10.1016/j.eswa.2016.09.006.

53. Verma H, Agrawal RK, Sharan A. An improved intuitionistic fuzzy c-means clustering algorithm incorporating local information for brain image segmentation. Applied Soft Computing. 2016;46:543–557. doi:https://doi.org/10.1016/j.asoc.2015.12.022.

54. Lei T, Jia X, Zhang Y, He L, Meng H, Nandi AK. Significantly fast and robust fuzzy c-means clustering algorithm based on morphological reconstruction and membership filtering. IEEE Transactions on Fuzzy Systems. 2018;26(5):3027–3041.

55. Pal NR, Bezdek JC. On cluster validity for the fuzzy c-means model. IEEE Transactions on Fuzzy systems. 1995;3(3):370–379.

56. Winkler R, Klawonn F, Kruse R. Fuzzy c-means in high dimensional spaces. International Journal of Fuzzy System Applications (IJFSA). 2011;1(1):1–16.

57. Zhang X, Jayavelu S, Liu L, Friedl MA, Henebry GM, Liu Y, et al. Evaluation of land surface phenology from VIIRS data using time series of PhenoCam imagery. Agricultural and Forest Meteorology. 2018;256:137–149.

58. Kalinicheva E, Ienco D, Sublime J, Trocan M. Unsupervised change detection analysis in satellite image time series using deep learning combined with graph-based approaches. IEEE Journal of Selected Topics in Applied Earth Observations and Remote Sensing. 2020;13:1450–1466.

59. Kalinicheva E, Sublime J, Trocan M. Neural network autoencoder for change detection in satellite image time series. In: 2018 25th IEEE International Conference on Electronics, Circuits and Systems (ICECS). IEEE; 2018. p. 641–642.

60. Kondmann L, Toker A, Saha S, Schölkopf B, Leal-Taixé L, Zhu XX. Spatial Context Awareness for Unsupervised Change Detection in Optical Satellite Images. arXiv preprint arXiv:211002068. 2021;.

61. Andresini G, Appice A, Iaia D, Malerba D, Taggio N, Aiello A. Leveraging autoencoders in change vector analysis of optical satellite images. Journal of Intelligent Information Systems. 2021;.

Fig1

Click here to access/download;Figure;Fig1.tif

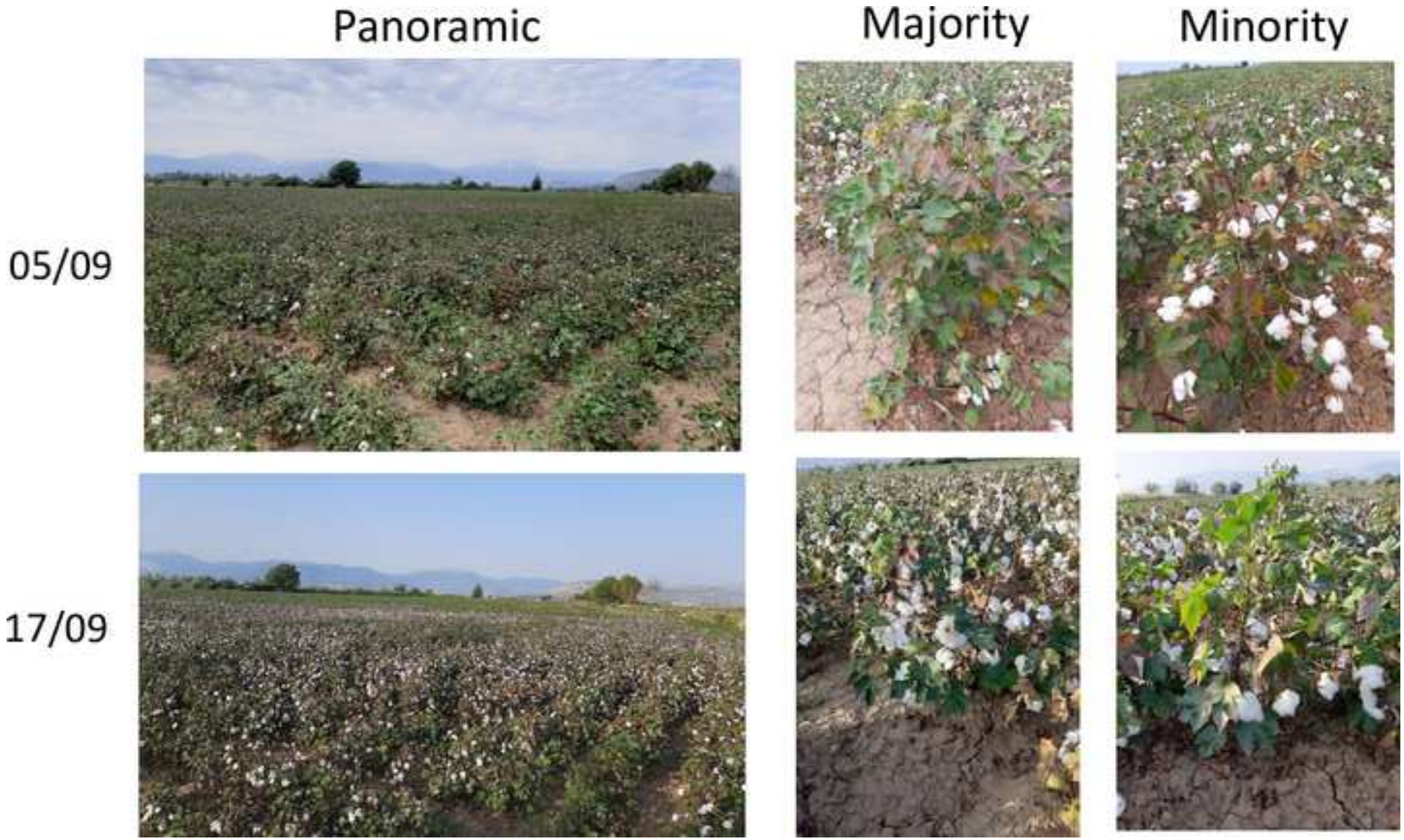

Fig2

Click here to access/download;Figure;Fig2.tif

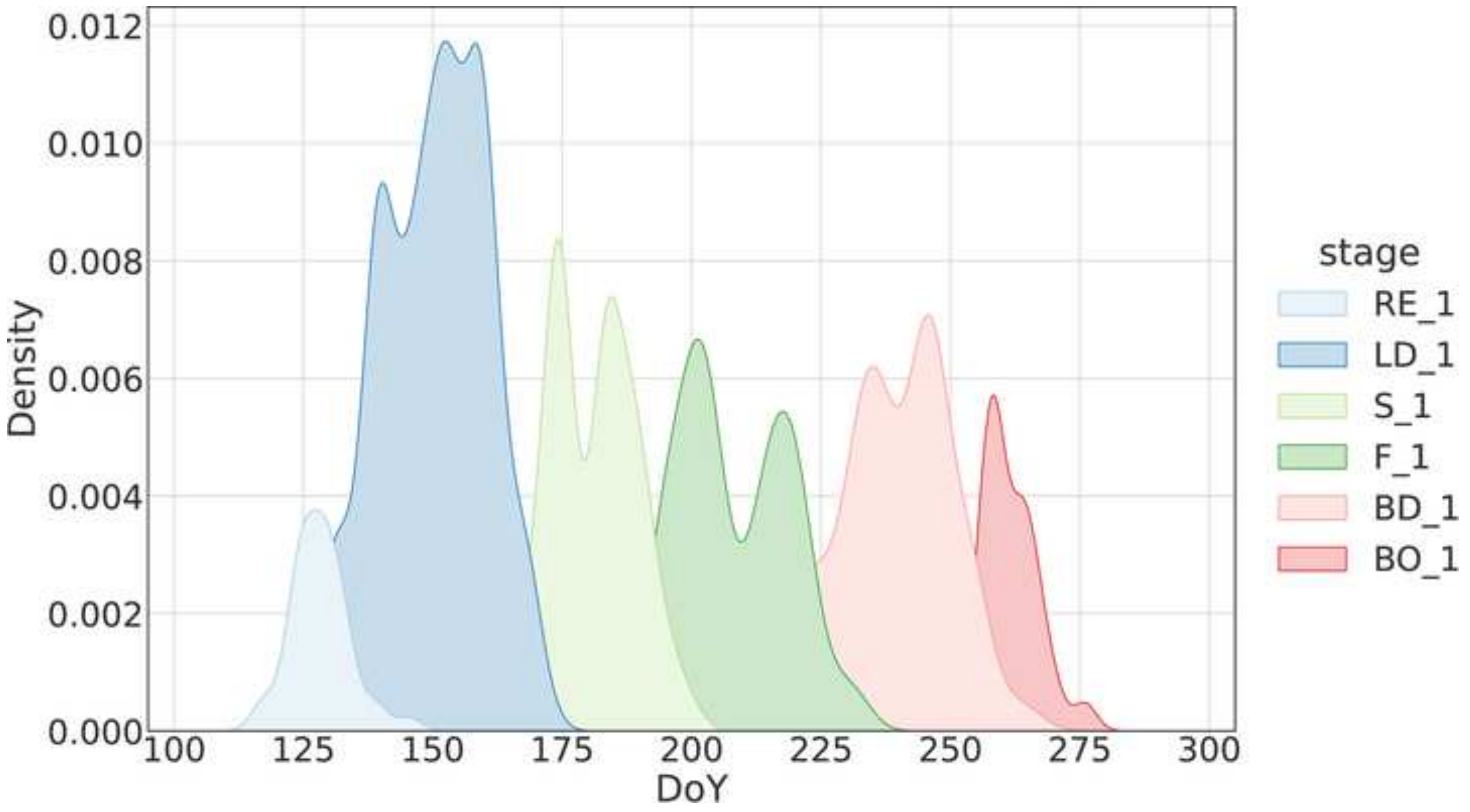

Fig3

Click here to access/download;Figure;Fig3.tif

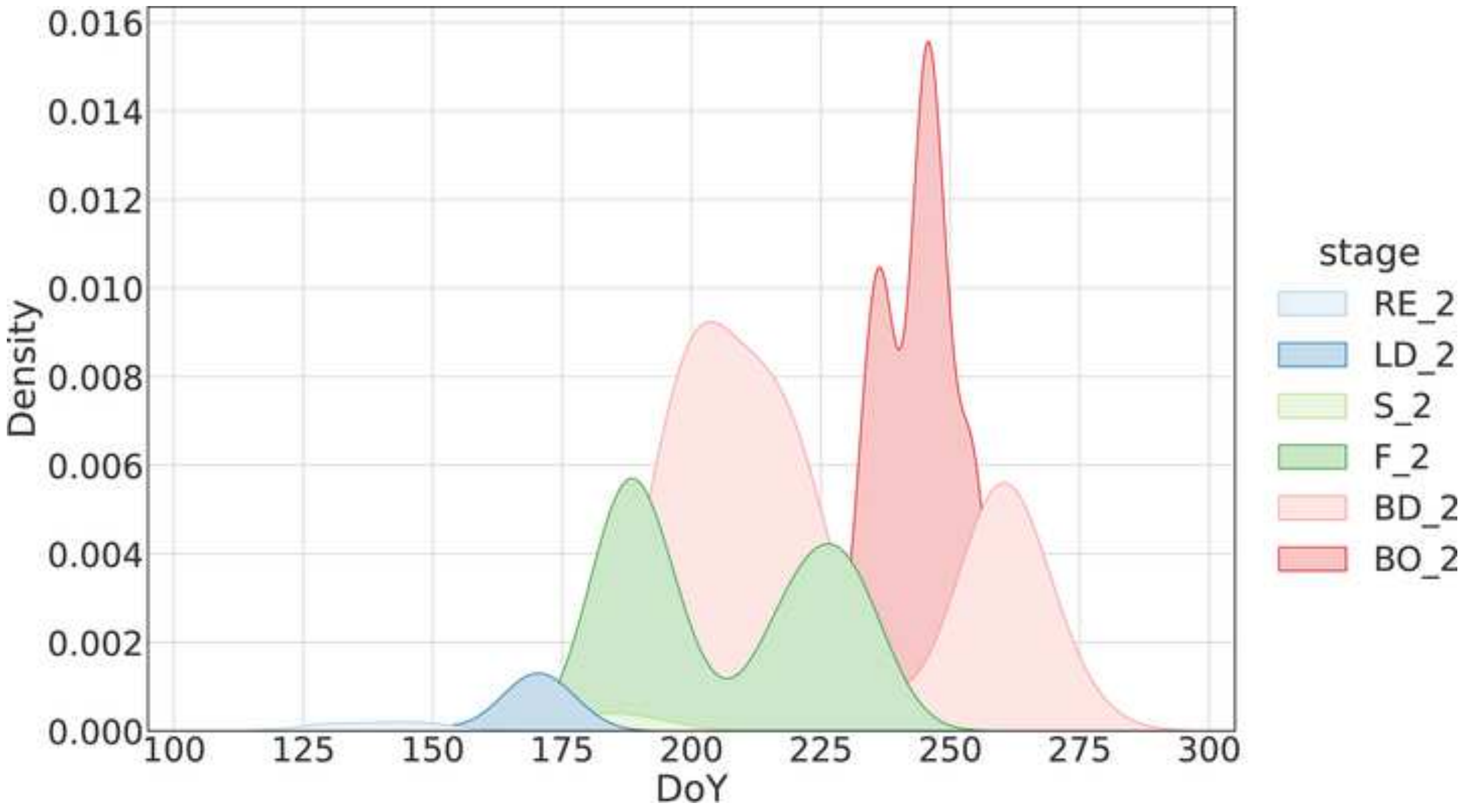

Fig4 Click here to access/download;Figure;Fig4.tif

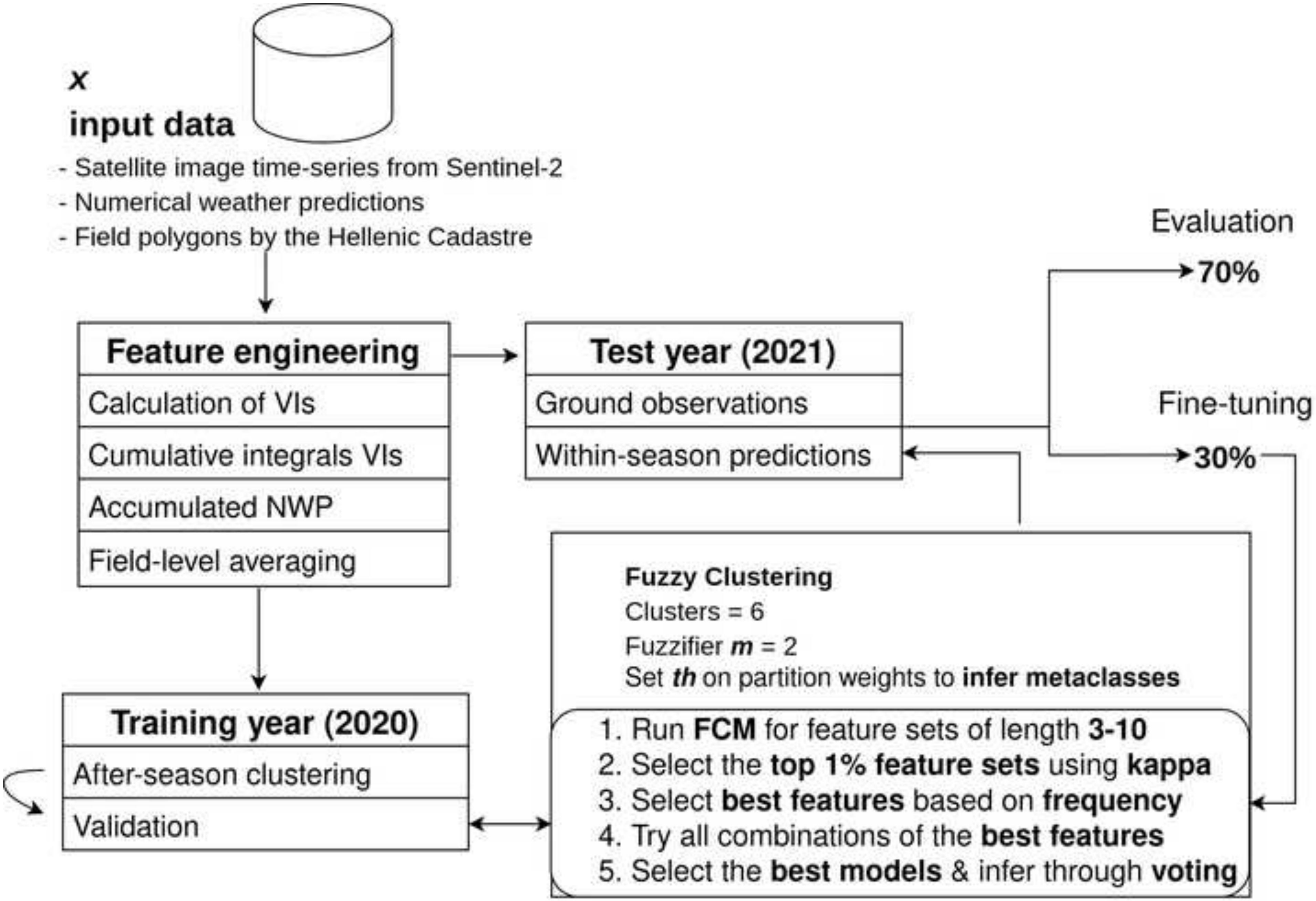

Fig5

Click here to access/download;Figure;Fig5.tif

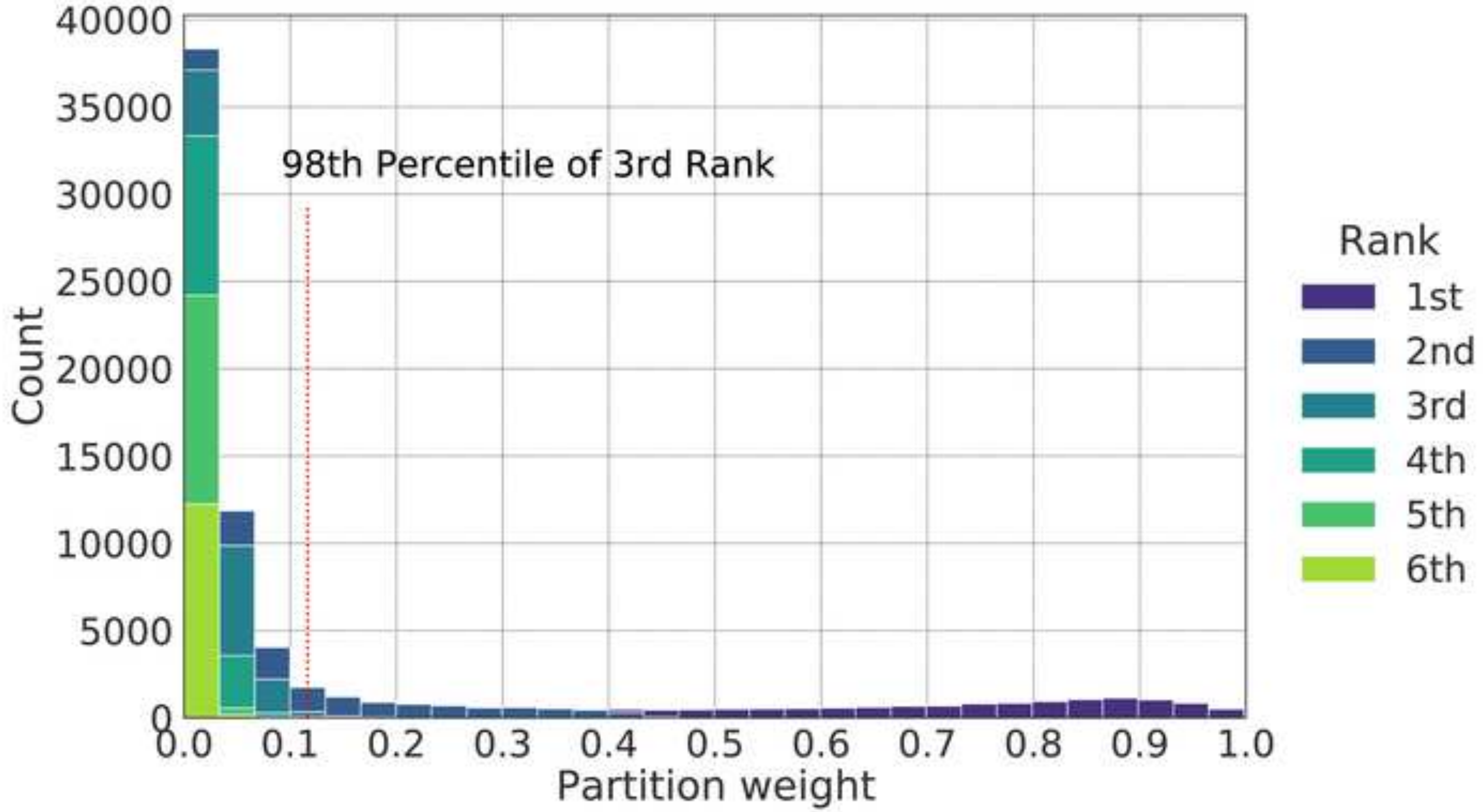

Fig6

Click here to access/download;Figure;Fig6.tif

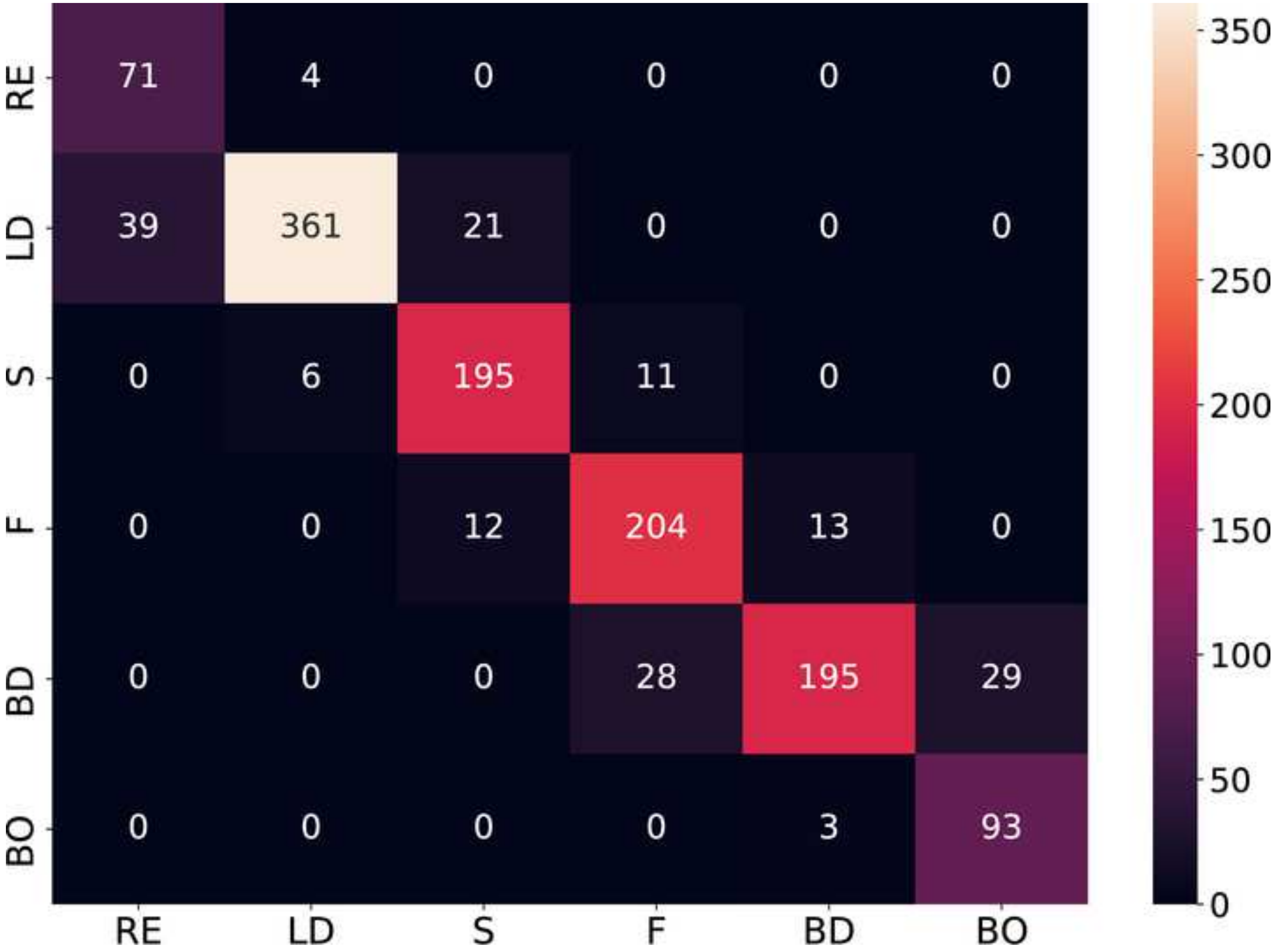

Fig7 Click here to access/download;Figure;Fig7.tif

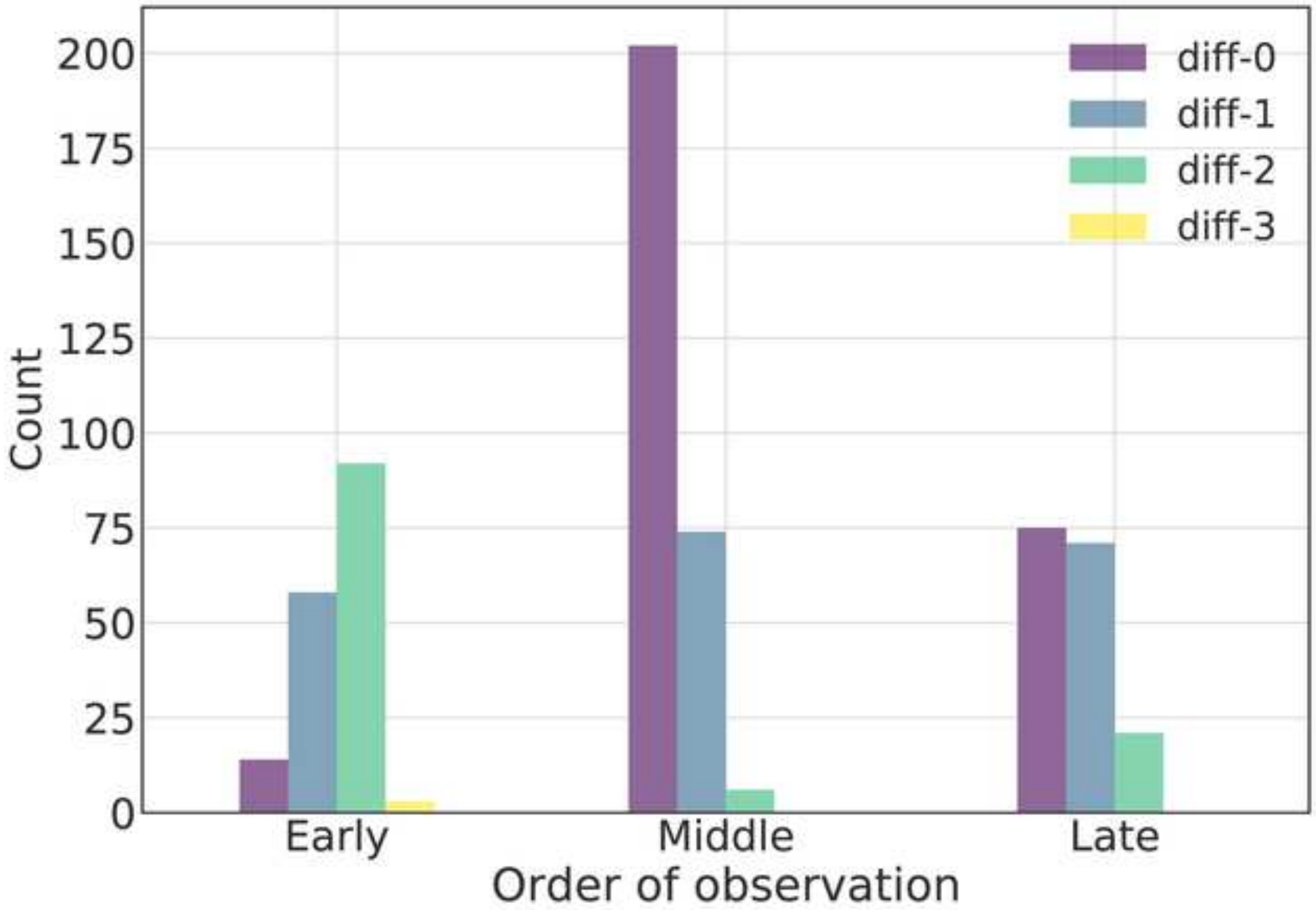

S1 table

Click here to access/download
Supporting Information
S1_table.pdf
